# Tailored structured peptide design with a key-cutting machine approach

**DOI:** 10.1038/s42256-025-01119-2

**Published:** 2025-10-21

**Authors:** Yan C. Leyva, Marcelo D. T. Torres, Carlos A. Oliva, Cesar de la Fuente-Nunez, Carlos A. Brizuela

**Affiliations:** 1 Computer Science Department, Center for Scientific Research and Higher Education at Ensenada (CICESE), Ensenada, Mexico; 2 Machine Biology Group, Departments of Psychiatry and Microbiology, Institute for Biomedical Informatics, Institute for Translational Medicine and Therapeutics, Perelman School of Medicine, University of Pennsylvania, Philadelphia, PA USA; 3 Departments of Bioengineering and Chemical and Biomolecular Engineering, School of Engineering and Applied Science, University of Pennsylvania, Philadelphia, PA USA; 4 Department of Chemistry, School of Arts and Sciences, University of Pennsylvania, Philadelphia, PA USA; 5 Penn Institute for Computational Science, University of Pennsylvania, Philadelphia, PA USA

**Keywords:** Computational models, Antimicrobials

## Abstract

Computational protein and peptide design is emerging as a transformative framework for engineering macromolecules with precise structures and functions, offering innovative solutions in medicine, biotechnology and materials science. However, current methods predominantly rely on generative models, which are expensive to train and modify. Here, we introduce the Key-Cutting Machine (KCM), an optimization-based platform that iteratively leverages structure prediction to match desired backbone geometries. KCM requires only a single graphics processing unit and enables seamless incorporation of user-defined requirements into the objective function, circumventing the high retraining costs typical of generative models while allowing straightforward assessment of measurable properties. By employing an estimation of distribution algorithm, KCM optimizes sequences on the basis of geometric, physicochemical and energetic criteria. We benchmarked its performance on α-helices, β-sheets, a combination of both and unstructured regions, demonstrating precise backbone geometry design. As a proof of concept, we applied KCM to antimicrobial peptide design by using a template antimicrobial peptide as the ‘key’, yielding a candidate with potent in vitro activity against multiple bacterial strains and efficacy in a murine infection model. KCM thus emerges as a robust tool for de novo protein and peptide design, offering a flexible paradigm for replicating and extending the structure–function relationships of existing templates.

## Abstract (web summary)

Powerful generative AI models for designing biological macromolecules are being developed, with applications in medicine, biotechnology and materials science, but these models are expensive to train and modify. Leyva et al. introduce the Key-Cutting Machine, an optimization-based platform for proteins and peptides that iteratively leverages structure prediction to match desired backbone geometries.

## Main

Protein and peptide design aims to construct synthetic proteins and peptides or modify existing ones to achieve new functionalities^1,2^. Successful designs include proteins that inhibit viral infections such as influenza^3,4^, SARS-CoV-2^5^ and HIV^6^, as well as enzymes that catalyse reactions for which no natural counterparts were previously known^7^. Additional applications include biosensors to detect various molecules^8^, including fentanyl^9^, self-assembling protein nanoparticle vaccines for SARS-CoV-2^10^, self-assembling nanomaterials^11^ and biological logic gates^12^. In the case of peptides, a simple generative model was proposed to design antimicrobial peptides against *Cutibacterium acnes*^13^. ProT-Diff^14^, another generative model, produced peptides; of these, 34 showed antibacterial activity against both Gram-positive and Gram-negative bacteria, as well as in vivo activity against a clinically relevant drug-resistant *Escherichia coli* strain. These advances highlight the broad therapeutic and biotechnological potential of computational protein and peptide design^15^.

Computational protein and peptide design is often framed as a combinatorial optimization problem, where the goal is to identify an amino-acid sequence that folds into a structure with minimal free energy^1^. This challenge is partially mitigated by discretizing side-chain conformations (rotamers)^16^. Thus, the task reduces to finding the optimal combination of rotamers that minimizes free energy for a given backbone. Alternative formulations include mixed-integer linear programming^17^ and cost function networks^18^. However, the protein design problem remains extraordinarily complex^19,20^.

Deep learning methods have emerged as powerful tools for protein design. Notable examples include ProteinMPNN^21^, a message-passing neural network, ProteinSolver^22^, a graph-based neural network, and ESM-IF1^23^, which integrates a geometric vector perceptron with a transformer. Given a structure in Protein Data Bank (PDB) format, these models predict the amino-acid sequences most likely to fold into the provided structure. Later methods improved upon ProteinMPNN and ESM-IF1 by enhancing sequence recovery and reducing perplexity through the incorporation of additional structural features. For example, SPDesign^24^ leverages structurally similar templates and builds a sequence profile to guide design. SPIN-CGNN^25^ computes distance-based contact maps and encodes them into a graph representation. PiFold^26^ extracts spatially invariant geometric features from atomic coordinates using a residue-level featurizer. These strategies support more accurate, flexible and context-aware sequence prediction.

Recently, generative models, including diffusion models, have been applied to protein design. These models, which include RFDiffusion^27^ and GRADE-IF^28^, can generate, from random noise, backbone structures that can then be paired with protein design networks (for example, ProteinMPNN) to produce corresponding sequences. Additionally, the integration of language model innovations has shown promise in diverse protein design tasks. For example, StructureGPT^29^ has been used to suggest sequence modifications that would improve solubility and structural stability. Similarly, TPGen^30^ has demonstrated the ability to generate short, diverse sequences that reliably fold into predefined secondary structures, as confirmed by molecular dynamics simulations. The main purpose of generative models is to explore new sequences and structures associated with new functions.

**Fig. 1:**
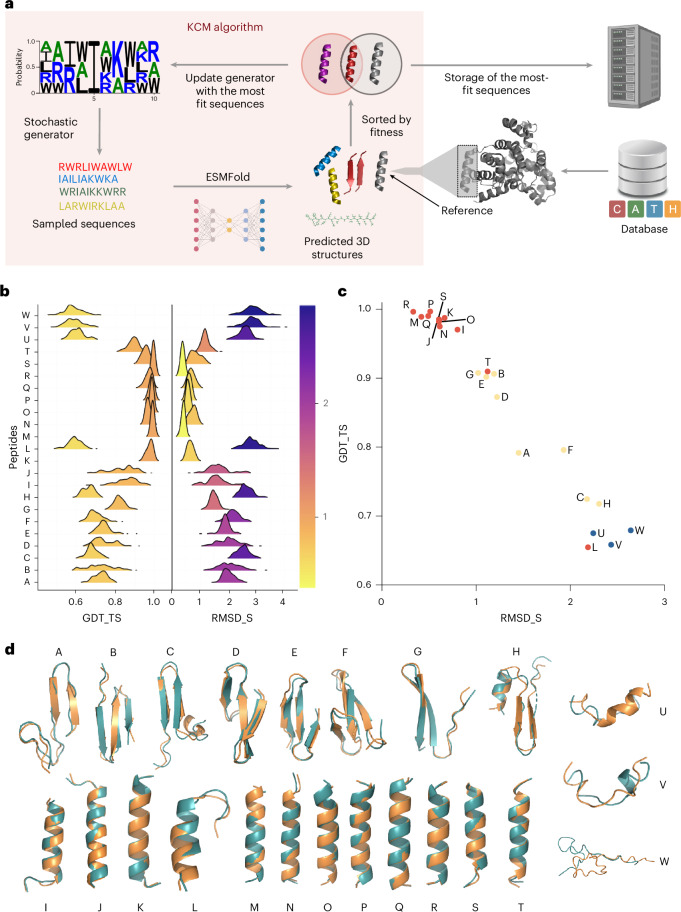
KCM algorithm. **a**, Schematic of the KCM algorithm including the stochastic generator and the three-dimensional (3D) structure predictor (ESMFold). **b**, Distribution for GDT_TS and RMSD_S from the best 100 designs according to their fitness, for each protein. Continuous colour range for both GDT_TS and RMSD_S. **c**, GDT_TS as a function of RMSD_S for the highest-fitness design of each protein. Mainly β-sheets in yellow, α-helix in red and those without defined secondary structure in blue. **d**, Backbone superposition of the reference protein (green) with respect to the backbone of the highest-fitness design (orange). Correspondence between characters and proteins for **b**–**d**: A, 1EMN; B, 1LQL; C, 1NP6; D, 1O6W; E, 2E45; F, 2KXQ; G, 1S7M; H, 1P9N; I, 5U1Y; J, 3CLQ; K, 3SB1; L, 2QQ8; M, 3M9Q; N, 3H25; O, 3EWK; P, 3C8V; Q, 2QIW; R, 2OAR; S, 2LKM; T, 1MSL; U, 3W68; V, 1R5L; W, 1N7D. Panel **a** created with BioRender.com. Source data

However, a major limitation of existing generative models is their substantial computational cost, primarily due to the need for retraining from scratch whenever a new measurable property is introduced into the loss function. For instance, training ESM-IF1 incurs a substantial computational cost. The model was trained on over 19,000 protein structures from the CATH 4.2 database (40% non-redundant) and supplemented with 12 million AlphaFold 2-predicted structures. Each epoch required 88 h on 32 graphics processing units (GPUs), totalling approximately 653 GPU-days. This level of resource consumption highlights the impracticality of retraining such models for iterative design tasks. To overcome this limitation, we propose an optimization-based model for protein and peptide design—referred to as the Key-Cutting Machine (KCM) model (Fig. 1a). KCM requires only a single GPU and enables the seamless incorporation of user-defined requirements into the objective function, thus avoiding the repeated retraining overhead typical of generative models and simplifying the assessment of measurable properties.

In this framework, the ‘key’ corresponds to the target structure and its sequence, and a structure-prediction method generates multiple copies of this key. KCM then refines these sequences by comparing the original key with its predicted copies. We implement an estimation of distribution algorithm (EDA)^31^ using an island model and employ ESMFold as the structure predictor to guide the optimization process. Importantly, the KCM algorithm does not require training, which allows it to be applied directly without the need for large training datasets or computationally expensive pretraining procedures. Other approaches that use evolutionary algorithms with a structure predictor in the loop address different design goals^32,33^.

As a proof of concept, we chose a 12-residue peptide with antimicrobial properties^34^. We computationally designed derivatives of this small peptide, synthesized them chemically and tested their antimicrobial activity both in vitro and in vivo.

## Results

Our methodology consists of three stages (Fig. 1). First, we define an optimization model for the KCM approach, characterized by an objective function to be maximized. Next, we propose an EDA to solve this model. Finally, we apply the algorithm to a dataset of proteins with known sequences and secondary structures, including α-helices, β-sheets and unstructured proteins. We also tested the KCM on proteins that are a combination of α-helices and β-sheets.

The protein design problem is daunting because of the immense sequence space and the unpredictable mapping from amino-acid sequences to structures^35^. Even a single amino-acid mutation can markedly alter the structure of a given protein or peptide. We hypothesize that our EDA can accurately estimate the distribution of structures within sequence space^36,37^.

### Structured peptide design: three types of secondary structure

In our experiments, protein designs dominated by α-helices required fewer generations to converge than their β-sheet counterparts, in part because α-helices are typically shorter. A termination condition of 100 generations was applied to α-helical proteins (for example, PDB identifiers (IDs) 5U1Y, 3CLQ, 3SB1, 2QQ8, 3M9Q, 3H25, 3EWK, 3C8V, 2QIW, 2OAR, 2LKM and 1MSL) and proteins lacking defined secondary structure (PDB IDs 3W68, 1R5L and 1N7D). For β-sheet proteins (PDB IDs 1EMN, 1LQL, 1NP6, 1O6W, 2E45, 2KXQ, 1S7M and 1P9N), we used a termination condition of 1,000 generations or until the global distance test total score (GDT_TS)^38^ reached at least 0.9. When the threshold was met earlier (for example, PDB IDs 1O6W, 2E45, 2KXQ, 1S7M and 1LQL), the algorithm was halted before 1,000 generations. For proteins with PDB IDs 1EMN, 1NP6 and 1P9N, the algorithm completed the full 1,000 generations (parameters are listed in Supplementary Table 1). For proteins with a combination of α-helices and β-sheets the termination criterion was a GDT_TS of 0.9 or higher or 350 generations, whichever came first.

In each generation, the number of objective function evaluations was calculated by multiplying the population size (five individuals) by the samples generated per individual (five) and the total number of islands (20), yielding 500 evaluations. In the 21st island, the individuals are no longer stochastic generators but amino-acid sequences, so we have 25 sequences in this island, requiring 25 evaluations, resulting in 525 objective function calls per generation.

For proteins primarily composed of α-helices, GDT_TS distributions trended toward higher values, approaching 1, whereas standard root mean square deviation (RMSD_S) distributions approached 0 (Fig. 1b and Supplementary Table 2). This pattern indicates high structural similarity and stability among the designs, with minimal variance. However, in proteins 5U1Y, 3CLQ and 2QQ8 (Fig. 1d, I, J and L), GDT_TS and RMSD_S distributions were more dispersed. This variance may stem from disordered regions at one terminus, which lack well-defined secondary structure and thus need more generations for convergence.

Proteins dominated by α-helices showed narrow GDT_TS s.d. values (0.00–0.07) yet larger RMSD_S variation (0.04–0.42) (Supplementary Table 2). Although higher RMSD_S variability helps prevent premature convergence, β-sheet proteins typically required longer runs and showed more structural diversity, evident in broader RMSD_S and GDT_TS distributions. Because β-sheet proteins had an average length of 32 residues—nearly double that of α-helical proteins (18 residues)—their search space was inherently more complex. Unstructured proteins posed even greater challenges for the algorithm.

We calculated the correlation between GDT_TS and RMSD_S for the top designs (Fig. 1c). Data points are colour-coded by secondary structure type: yellow (β-sheet), red (α-helix) or blue (unstructured). Red points cluster toward higher GDT_TS and lower RMSD_S values, whereas blue points show lower accuracy. Protein 2QQ8 (L), which contains an unstructured region, stands out with lower GDT_TS and RMSD_S scores compared with other α-helical proteins.

Superimposing the highest-fitness designs onto their reference structures (Fig. 1d) reveals notable structural similarity, particularly in α-helical and β-sheet regions. Proteins with unstructured segments showed lower similarity, although extended runs did improve alignment. Notably, the highest sequence identity between the best designs and the reference sequences was merely 24%, with an average of 11% (Supplementary Table 2), suggesting that the algorithm can converge on structurally similar solutions despite low sequence identity.

On this same dataset of 23 proteins, using a Bayesian ranking^39^, we compared KCM with three well-known generative models: ProteinMPNN, ESM-IF1 and ProteinSolver. For a given target backbone, ProteinMPNN generated 256 sequences in four runs, while ESM-IF1 and ProteinSolver each produced 250 sequences. For each target, we selected the sequence with the highest GDT_TS and the sequence with the lowest RMSD for comparison. For KCM, we likewise chose the best sequences according to each criterion. For a fair comparison, all solutions from each algorithm were sorted on the basis of the fitness function used in our algorithm. When examining only 50 solutions (Supplementary Fig. 1a–f and Supplementary Table 3), KCM surpassed the other approaches in RMSD but lagged behind ESM-IF1 and ProteinMPNN in GDT_TS. When 250 solutions were considered (Supplementary Fig. 1g–l and Supplementary Table 4), KCM again outperformed all other methods in RMSD but fell short of ESM-IF1 in GDT_TS. Note that these proteins might have been used in the generative models’ training process. In this case, of the 23 proteins tested, only two (PDB IDs 1P9N and 5U1Y) were not included in CATH 4.2, from which the training sets of ProteinMPNN and ESM-IF1 were taken.

To analyse the performance of KCM when proteins with a combination of α-helix and β-sheet are included, eight new designs were undertaken (Supplementary Table 5); the results are reported in Supplementary Table 6. Here six out of eight designs achieved a GDT_TS larger than 0.9; this is better than the case for β-sheet-only designs, where only four out of eight achieved a GDT_TS larger than 0.9, even when more computation effort was allowed. The superpositions of the targets and their corresponding designs are shown in Supplementary Fig. 2.

To estimate the cost of designing longer sequences, we evaluated the structure prediction time of ESMFold on randomly generated sequences ranging from 10 to 400 residues. Predictions for sequences up to 100 residues required less than 2 s each, while sequences of 400 residues required approximately 10 times more computation time (Supplementary Fig. 3). The algorithm was also applied to design two proteins of 100 residues each, PDB IDs 2F77 and 2HLQ. After 2,000 generations the GDT_TS reached 0.38 for both targets, showing a limitation in designing proteins of this size without parameter tuning.

### Antimicrobial peptide design

As a proof of concept, we selected IDR-2009, a 12-residue peptide (sequence KWRLLIRWRIQK) known for its potent antimicrobial activity^34^. This peptide was chosen for practical reasons: its antimicrobial activity can be readily validated in vitro and short sequences are amenable to synthesis. Additionally, KCM performed well on α-helices (Supplementary Table 2). We tested multiple objective function configurations to evaluate whether KCM could generate variants with favourable solubility and synthetic feasibility.

We did not assume that the peptide’s function directly depends on its geometry. Instead, we investigated whether mirroring its predicted backbone and its amino-acid properties and minimizing its energy would yield sequences with comparable structure and properties.

To design a set of peptides using the IDR-2009 backbone as a template, we proceeded as follows. (1) We obtained the three-dimensional structure of the peptide using the Google Colab version of AlphaFold 2^40,41^, on the basis of its sequence. Within AlphaFold 2, we used the default parameters but increased to 48 recycles and three relaxation iterations. Even though this implementation of AlphaFold 2 does not use structural templates and uses a limited-size database to construct the multiple sequence alignment, the resulting average predicted local distance difference test was higher than 0.8, which is considered a confident predicted local distance difference test score^42,43^. This resulting backbone structure served as the input for our KCM. (2) We conducted the design under four different schemes, primarily varying the protein similarity function. The four schemes were the following.

#### All terms included (AT)

All terms in equation (1) were applied.

1 $$\begin{array}{l}f=\left[\displaystyle\frac{1}{1+{\mathrm{RMSD}}\_{\mathrm{S}}}+{\mathrm{GDT}}\_{\mathrm{TS}}+\frac{1}{1+{\mathrm{RMSD}}\_{\mathrm{DM}}}\right]\\\qquad\times\left[1+\displaystyle\frac{1}{3\exp\left(\displaystyle\frac{{E}_{\mathrm{d}}+30}{30}\right)}\right]+\displaystyle\frac{1}{1+\underline{\mathrm{KL}}}\end{array}$$

where KL is the mean Kullback–Leibler divergence (Supplementary equation (14), see details in Supplementary Section 1.7) of the descriptors computed from the sequence. RMSD_S, GDT_TS and RMSD_DM are also defined in Supplementary Information, in Sections 1.1, 1.3 and 1.4, respectively. The energy of the designed sequence (*E*_d_) term is calculated by applying the Rosetta energy function to the structure predicted by ESMFold for each designed sequence.

#### No descriptors included (ND)

We excluded the KL divergence terms from the objective function:

2 $$\begin{array}{l}f=\left[\displaystyle\frac{1}{1+{\mathrm{RMSD}}\_{\mathrm{S}}}+{\mathrm{GDT}}\_{\mathrm{TS}}+\displaystyle\frac{1}{1+{\mathrm{RMSD}}\_{\mathrm{DM}}}\right]\\\qquad\times \left[1+\displaystyle\frac{1}{3\exp \left(\displaystyle\frac{{E}_{\mathrm{d}}+30}{30}\right)}\right].\end{array}$$

#### No geometry terms included (NG)

We excluded the geometric similarity criteria from the objective function and weighted the energy terms negligibly:

3 $$\begin{array}{l}f=\left[\displaystyle\frac{1}{1+{\mathrm{RMSD}}\_{\mathrm{S}}}+{\mathrm{GDT}}\_{\mathrm{TS}}+\displaystyle\frac{1}{1+{\mathrm{RMSD}}\_{\mathrm{DM}}}\right]\\\quad\times\left[1+\displaystyle\frac{1}{3\exp\left(\displaystyle\frac{{E}_{\mathrm{d}}+30}{30}\right)}\right]+\displaystyle\frac{1}{1+\underline{\mathrm{KL}}}\end{array}$$

where RMSD_S = RMSD_DM = 10^6^ and GDT_TS = 0.

Notice that the difference between equations (3) and (1) is that in equation (3) the geometric term is neglected and therefore so is the energy term, the descriptor difference remaining as the main component in the objective function, while in equation (1) all terms are taken into account.

#### No energy terms included (NE)

We omitted the energy term from the objective function:

4 $$f=\left[\frac{1}{1+{\mathrm{RMSD}}\_{\mathrm{S}}}+{\mathrm{GDT}}\_{\mathrm{TS}}+\frac{1}{1+{\mathrm{RMSD}}\_{\mathrm{DM}}}\right]+\frac{1}{1+\underline{\mathrm{KL}}}.$$

For each scheme, we generated a set of sequences and ranked them in descending order according to their fitness (Supplementary Table 7 lists the evolutionary algorithm parameters). We then analysed these ranked sequences with AMPDiscover^44^ to predict their potential antimicrobial activity. We further examined the top 10 sequences from each scheme (Supplementary Tables 8–10).

### In vitro antimicrobial activity of peptides

**Fig. 2:**
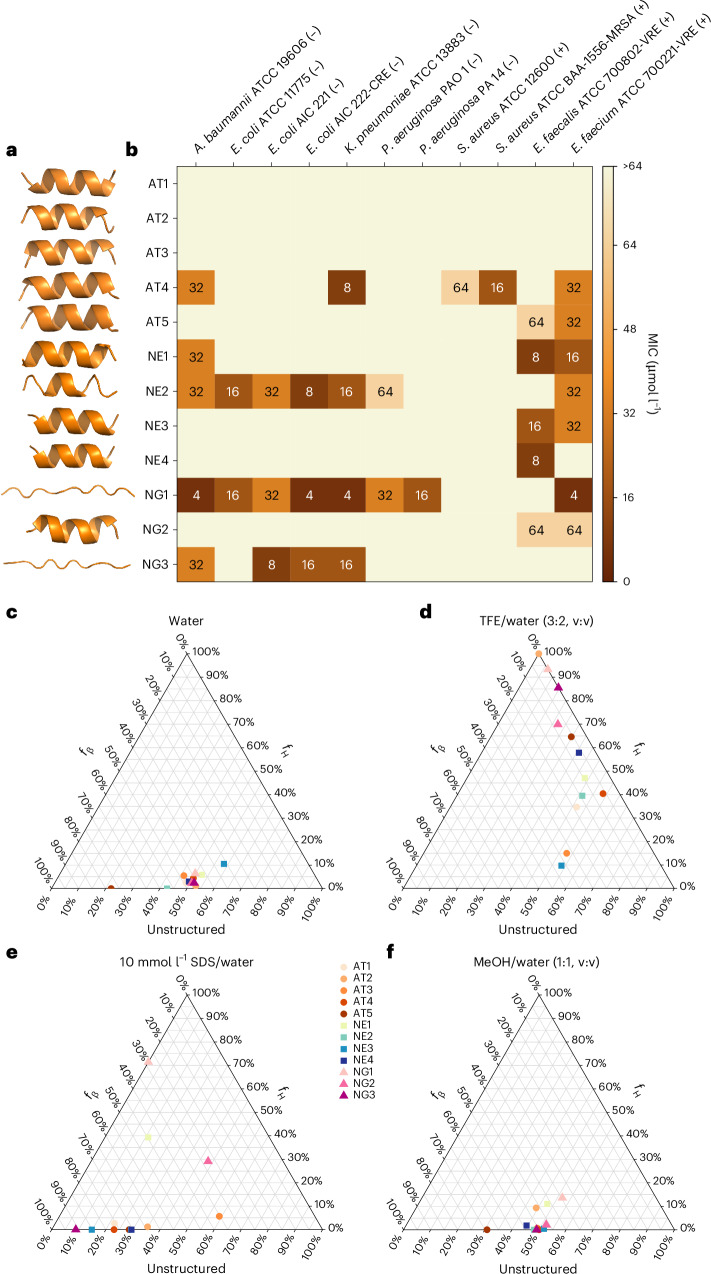
Antimicrobial activity and secondary structure of computationally designed peptides. **a**, Structure prediction of the synthesized peptides. **b**, Heatmap of the antimicrobial activities of the peptides against 11 clinically relevant pathogens, including four strains resistant to conventional antibiotics. Briefly, 10^6^ bacterial cells and serially diluted peptides (1-64 μmol l^−1^) were incubated at 37 °C. One day after treatment, the optical density at 600 nm was measured in a microplate reader to evaluate bacterial growth in the presence of the peptides. MIC values in the heatmap are the mode of the replicates in each condition. **c**–**f**, Ternary plots showing the percentage of secondary structure for each peptide (at 50 μmol l^−1^) in four different solvents: water (**c**), 60% TFE in water (**d**), 10 mmol l^−1^ SDS in water (**e**) and 50% methanol (MeOH) in water (**f**). Secondary structure fractions were calculated using the BeStSel server^65^. Source data

We synthesized 12 sequences selected for their ease of synthesis and low aggregation potential: five from AT, four from NE and three from NG. We excluded the ND group because its peptides were challenging to synthesize and solubilize. We determined their minimum inhibitory concentrations (MICs) against 11 clinically relevant strains, including the ESKAPEE pathogens. Nine of the 12 peptides (75%) exhibited MIC values of ≤64 μmol l^−1^ against at least one strain (Fig. 2a,b), surpassing hit rates from many existing machine learning/deep learning methods. The three inactive peptides (AT1, AT2, AT3) belonged to the AT group, had low net charge (<2) and displayed lower normalized hydrophobicity. This indicates that incorporating all criteria into the objective function might increase the complexity of the search landscape and hinder the discovery of highly active sequences. Further investigation is needed to confirm this hypothesis.

### Secondary structure of designed peptides

Because the peptides were designed using different criteria, we sought to assess their secondary-structure tendencies. To evaluate secondary structure, we subjected the peptides to four different media: water (Fig. 2c and Supplementary Fig. 4a, e), a helix-inducing medium (trifluoroethanol (TFE) in water, 3:2, v:v; Fig. 2d and Supplementary Fig. 4b, e), a membrane-mimicking environment (SDS at 10 mmol l^−1^; Fig. 2e and Supplementary Fig. 4c, e) and a β-inducer medium (methanol in water, 1:1, v:v; Fig. 2f and Supplementary Fig. 4d, e).

When geometric similarity criteria were omitted and energy terms were minimally weighted (NG group), the designed peptides displayed greater structural plasticity. They were initially unstructured forms in water and the methanol/water mixture. However, they adopted highly helical conformations in the TFE/water mixture and predominantly β-like conformations in the membrane-mimicking medium. Peptides from the AT and NE groups remained unstructured in water yet adopted mostly β-like conformations in SDS and the methanol/water mixture. Notably, in the presence of SDS micelles, NE1 showed an equal proportion of β-like and helical structures.

Among the peptides, AT2, AT5 and NE4 formed the most helical conformations in the TFE/water mixture, despite being among the least amphiphilic peptides. Conversely, AT3 and NE3, which were among the more amphiphilic peptides, predominantly retained a β-like structure even in the helix-inducing medium. No clear trend emerged linking secondary structure directly to antimicrobial activity.

### Mechanism of action

**Fig. 3:**
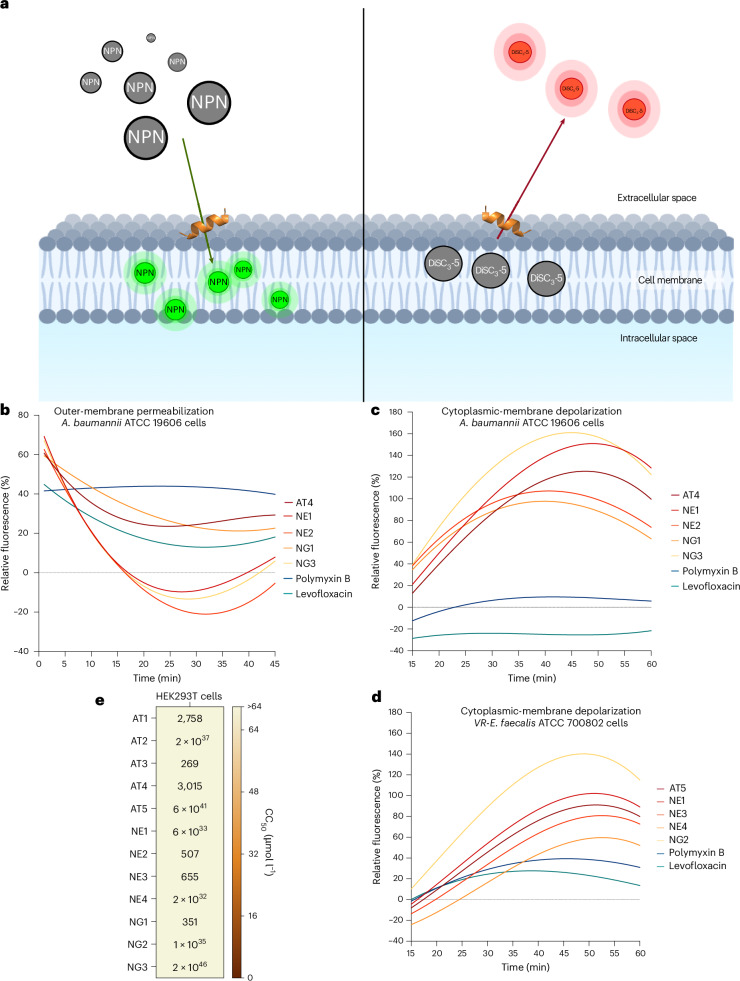
Mechanism of action and cytotoxic activity of designed peptides. **a**, To assess whether the peptides act on bacterial membranes, all active peptides against *A. baumannii* American Type Culture Collection (ATCC) 19606 were subjected to outer-membrane permeabilization, and peptides active against *A. baumannii* ATCC 19606 and vancomycin-resistant *E. faecalis* ATCC 700802 were tested in cytoplasmic-membrane depolarization assays. **b**, The fluorescent probe NPN was used to assess membrane permeabilization induced by the tested peptides. **c**,**d**, The fluorescent probe DiSC_3_-5 was used to evaluate membrane depolarization caused by the designed peptides in *A. baumannii* ATCC 19606 (**c**) and vancomycin-resistant *E. faecalis* ATCC 700802 (**d**). The values displayed represent the relative fluorescence of both probes, with nonlinear fitting compared with the baseline of the untreated control (buffer + bacteria + fluorescent dye) and benchmarked against the antibiotics polymyxin B and levofloxacin. **e**, Cytotoxic concentrations leading to 50% cell lysis (CC_50_) were determined by interpolating the dose–response data using a nonlinear regression curve. All experiments were performed in three independent replicates. Panel **a** created with BioRender.com. Source data

We next explored the membrane-related mechanism of action of a subset of peptides (AT4, NE1, NE2, NG1, NG3) against *Acinetobacter* *baumannii* and vancomycin-resistant *Enterococcus faecalis* (Fig. 3a). Using *N*-phenyl-1-naphthylamine (NPN) uptake assays (Fig. 3b and Supplementary Fig. 5a) and membrane depolarization assays with 3,3′-dipropylthiadicarbocyanine iodide (DiSC_3_-5) (Fig. 3c, d and Supplementary Fig. 5b, c), we found that all peptides disrupted the bacterial membrane potential to varying degrees. NE1 and NG3 were particularly effective at depolarizing the cytoplasmic membrane of *A. baumannii*. Although none outperformed polymyxin B in outer-membrane permeabilization, several peptides matched levofloxacin’s effects, underscoring their potential as membrane-targeting agents.

### Cytotoxicity assays

Cytotoxicity was evaluated using human embryonic kidney (HEK293T) cells (Fig. 3e)^45^. None of the 12 peptides caused substantial cytotoxicity at the tested concentrations (4–64 μmol l^−1^), contrasting with the parental IDR-2009 peptide, which did show cytotoxicity^34^. The lowest cytotoxic concentration leading to 50% cell lysis among inactive peptides (AT3) was 269 μmol l^−1^, indicating a favourable therapeutic window.

### Anti-infective efficacy in animal models

**Fig. 4:**
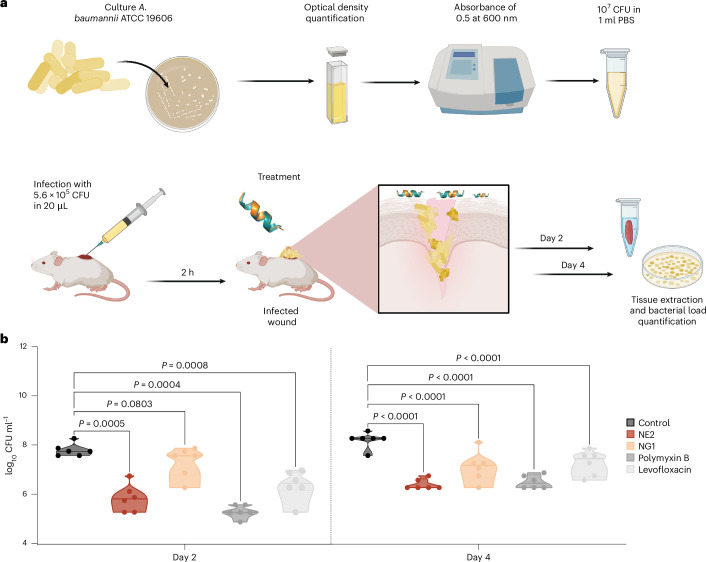
Anti-infective activity of designed peptides in a skin scarification animal model. **a**, Schematic representation of the skin abscess mouse model used to assess the anti-infective activity of the designed peptides (*n* = 6) against *A. baumannii* ATCC 19606. CFU, colony-forming units. **b**, NE2 and NG1, administered at their MIC (32 and 4 μmol l^−1^, respectively) in a single dose post-infection, inhibited the proliferation of the infection for up to 4 d after treatment compared with the untreated control group. Notably, NE2 reduced the infection in some mice, demonstrating activity comparable to the control antibiotics, polymyxin B and levofloxacin. Statistical significance in panel **b** was determined using one-way ANOVA followed by Dunnett’s test; *P* values are shown in the graphs. In the violin, the centre line represents the mean, the box limits the first and third quartiles and the whiskers (minima and maxima) represent 1.5 × the interquartile range. Panel **a** created with BioRender.com. Source data

We tested NE2 and NG1 in two murine models: skin abscess^46–49^ (Fig. 4) and a deep thigh infection^50–52^ (Supplementary Fig. 6c–e). In both models, the peptides reduced *A. baumannii* bacterial loads by up to two orders of magnitude, comparable to the last-resort antibiotic polymyxin B and to our other antibiotic control levofloxacin. No weight loss or skin damage was observed, indicating good tolerability. These results confirm that KCM-designed peptides can achieve clinically relevant anti-infective efficacy.

## Discussion

This study introduces and validates the KCM approach for computational structured peptide design. In contrast to purely generative frameworks, which use trained models to rapidly generate large numbers of candidate sequences, KCM iteratively refines candidate sequences through structure prediction and optimization. The results observed with mixed secondary structures such as α-helices and β-sheets support the idea that the α-helices are easier to design than β-sheets; when we have only β-sheets we have a lower success percentage (50%, Supplementary Table 2) than when we design α-helices and β-sheets (success percentage of 75%, Supplementary Table 5). When we have only α-helices, KCM achieves 91.6% success (Supplementary Table 2). Here, designs that achieved GDT_TS of 90% or more are considered successful. Overall, our results demonstrate that KCM can converge on sequences whose backbone geometries strongly resemble those of target proteins, spanning α-helices, β-sheets, combinations of α-helices and β-sheets, and unstructured regions, even when sequence identity is low. This suggests that KCM effectively explores the structural landscape by continuously updating the distribution of candidate sequences on the basis of both geometric and physicochemical criteria. However, without a proper parameter tuning, KCM was limited when designing proteins composed of 100 amino-acid residues or larger.

A key advantage of KCM is its ability to seamlessly integrate a wide range of objective functions beyond backbone geometry. In particular, it can incorporate Kullback–Leibler divergence or Jeffrey’s distance for amino-acid descriptors, as well as energy terms and other chemically relevant properties. This flexibility allows researchers to add or remove objectives—such as solubility, stability or functional motifs—within a single GPU environment, eliminating the need to retrain a deep learning model when new properties are introduced into the loss function. In contrast to generative models that require substantial computational resources for retraining, KCM offers an adaptable, resource-efficient solution for computational protein design.

Another key advantage of the proposed KCM framework lies in its interpretability and accessibility. Unlike deep learning-based generative models, KCM does not require a training phase; instead, it uses an explicitly defined objective function to guide the design process, enabling users to control and tailor design preferences directly. In addition, KCM employs an island-based evolutionary paradigm that is inherently parallelizable. While the current implementation operates on a single GPU, each island can, in principle, be executed independently across multiple processors or GPUs. This conceptual scalability allows KCM to benefit from parallel environments when available, while remaining efficient and accessible on modest hardware.

Beyond benchmarking KCM on proteins of diverse secondary structure, we applied it to the design of antimicrobial peptides. Despite not explicitly encoding direct activity constraints into our fitness function, the KCM-derived peptides retained antimicrobial activity that was comparable to or exceeded many machine learning/deep learning-based designs reported in the literature^13,14^. Several designs also exhibited lower cytotoxicity than the parental antimicrobial peptide (IDR-2009), demonstrating that KCM can discover sequences with improved therapeutic windows.

Structural analyses revealed that relaxing energy and geometric similarity constraints leads to higher structural plasticity, with designed sequences frequently transitioning between helical and β-like conformations depending on environmental factors. Notably, this plasticity did not necessarily reduce antimicrobial activity, suggesting that multiple structural pathways can yield effective membrane-disrupting peptides. These outcomes highlight the need to balance structural and energetic constraints with functional sequence-space exploration.

In summary, KCM provides an alternative paradigm for peptide and protein design, combining the adaptability of an iterative, optimization-based framework with the power of modern structure-prediction tools. Its demonstrated success in designing peptides with potent antimicrobial activity and reduced toxicity highlights the method’s potential in therapeutic development. Future work may explore more sophisticated descriptors—such as immunogenicity, proteolytic stability and post-translational modifications—as well as advanced multi-objective strategies and protein–water interactions, especially in the case of intrinsically disordered proteins, to enhance both throughput and exploration of sequence space. Ultimately, the KCM model represents a promising step toward more versatile and customizable computational design methodologies for proteins and peptides.

### Limitations of the study

Despite these advantages, several challenges remain with the KCM model. Its reliance on iterative structure prediction makes it more computationally intensive than standard pretrained generative approaches, such as ProteinMPNN or ESM-IF1, which can complete designs in seconds. For example, designing an α-helical sequence in our benchmark set requires about 12 h on a single GPU (RTX 3090 Ti) coupled with an Intel i5 CPU with 32 GB of random-access memory. This task is particularly challenging for short, dynamic peptides, which may adopt multiple conformations in solution. Integrating ensemble-based structure prediction or incorporating biophysical data (for example, nuclear magnetic resonance) could improve accuracy for more flexible targets. KCM is currently limited for larger proteins (100 amino-acid residues or larger), because population size and number of generations will need to increase considerably. This limits KCM’s current design capability to structured peptides.

Nonetheless, a key benefit of the KCM framework is that it does not require retraining. For instance, incorporating new design constraints or targeting a different functionality in a traditional generative model typically necessitates costly model retraining. In contrast, KCM enables these changes to be implemented directly by modifying the objective function, eliminating the need for reoptimization of the generative backbone. This affords greater flexibility in exploring diverse design criteria. While repeated structure predictions introduce computational overhead, this cost may be mitigated in future iterations through parallelization strategies or the integration of faster structure predictors.

Additionally, a single key (design target) was used to generate copies (peptide variants) for experimental testing. Further validation is needed to fully establish the generalizability of the proposed method for functional design.

## Methods

### Objective function

The objective function that we propose in this work for the key-cutting machine approach consists of a combination of different similarity measures between the structure, energy and chemical/evolutionary descriptors of a designed protein and those of the target protein. To measure structural similarity, we used the RMSD_S^53,54^, the GDT_TS^38^, the template modelling score (TM_S)^55^ and the RMSD_DM. The Rosetta energy function REF15^56^ is used to measure the energy of a protein structure. Finally, the chemical descriptors computed consisted of a subset of the iLearn protein descriptors (Supplementary Table 11)^11^ and the decoded vector representation from the ESM-2 language model^57^. Given the descriptor vectors for a model’s sequence and the reference’s sequence, the similarity between them can be computed by using Jeffrey’s distance^58^ (described in detail in Supplementary Section 1).

Notice that all these criteria measure the similarity or difference between a particular aspect of a designed protein and the reference protein. For example, RMSD_S, TM_S, GDT_TS and RMSD_DM all measure structural similarity. In this case, the structure of a designed sequence is predicted using ESMFold, and the resulting structure is then superimposed with the reference structure. On the other hand, Jeffrey’s distance between ESM-2 and iLearn descriptor vectors measures how similar the two proteins are in terms of both iLearn and ESM-2 descriptors.

The objective function to be maximized by our protein design algorithm is given as

5 $$f={f}_{1}+{f}_{2}+{f}_{3}$$

where

$$\begin{array}{c}{f}_{1}=\displaystyle\frac{1}{1+{\mathrm{RMSD}}\_{\mathrm{S}}}+{\mathrm{GDT}}\_{\mathrm{TS}}+\displaystyle\frac{1}{1+{\mathrm{RMSD}}\_{\mathrm{DM}}}+{\mathrm{TM}}\_{\mathrm{S}}\\ {f}_{2}=\displaystyle\frac{{f}_{1}}{4}\displaystyle\frac{1}{\exp \left(\displaystyle\frac{|{E}_{\mathrm{r}}-{E}_{\mathrm{d}}|}{\omega }\right)}\\ {f}_{3}=\displaystyle\frac{2}{1+\displaystyle\frac{1}{10}\mathop{\sum }\nolimits_{i=1}^{10}{D}_{\mathrm{J}}^{i}}\end{array}$$

and *E*_d_ and *E*_r_ are, respectively, the energy values of the model and reference structures, both calculated using the Rosetta energy function. Also, for all 1 ≤ *i* ≤ 10, the symbol ${D}_{\mathrm{J}}^{i}$ denotes Jeffrey’s distance, computed from the *i*th descriptor vector of both the model and the reference sequences. Note that the number of such vectors for each protein is ten, where nine are computed with iLearn and one with ESM-2. Finally, *ω* ∈ *R* is a parameter for contracting or dilating the exponential function.

The objective function is unaffected by rotations and translations of the structure, since both the model and reference structures are superimposed before calculating all criteria. Also, each of the terms *f*_1_, *f*_2_ and *f*_3_ contributes with a different magnitude to the total value of *f*. Since the maximum value given by the said function is 7, *f*_1_ represents four-sevenths of the total value, while *f*_2_ and *f*_3_ represent one-seventh and two-sevenths of the total, respectively. The weights for *f*_1_, *f*_2_ and *f*_3_ were empirically determined; identifying the optimal weighting for each component of the objective function remains an important direction for future work. Function *f* will also have values close to zero when the model and reference proteins are different in all the criteria considered.

An important advantage of KCM is its ability to seamlessly incorporate user-defined constraints. For example, if a user wishes to enforce the presence of hydrophobic amino-acid residues at specific positions (for example, positions *i* and *j*), the sequence generators (Supplementary Section 2) can be configured to assign zero probability to amino-acid residues that do not meet this criterion at those positions. Additionally, if the user aims to favour properties such as solubility or reduced aggregation, machine learning-based predictors can be integrated to define additional terms in the objective function, as demonstrated in a related approach^59^.

### KCM algorithm

The algorithm follows the island paradigm^60^, and in total there are 21 islands. The islands evolve synchronously and within each island we have a general procedure (Fig. 1). The first component is a stochastic sequence generator (structure of the individuals in the population), which depends on the mathematical model designed to learn how highly fit sequences are distributed within the feasible solution space. That is, each individual in the population encodes a probability distribution over amino acids at each position in the sequence, on the basis of one of six stochastic generator models. Sequences are sampled from these distributions and evaluated using the objective function, with top performers retained for the next generation. Details of the six generator models are provided in Supplementary Table 12. The second component is a three-dimensional structure predictor; in this case, ESMFold was used. It was chosen over the more accurate and confident AlphaFold 2 due to its notably faster prediction. The third component is the objective function, which is responsible for determining the similarity between the designs and the reference protein (the key). Finally, the fourth module is a repository where the top *t* sequences generated within the islands are stored according to their fitness.

The island model is equipped with a communication strategy. At the end of each generation, with probability *P*, two islands can collaborate, and the information shared is through the sequences. Each island stores the top *t* sequences with the highest fitness obtained throughout all generations of the algorithm. With probability proportional to their fitness, *m*_1_ sequences are selected from the *t* stored in the first island. All selected sequences that surpass the fitness of the weakest sequences in the second island will replace them. The same procedure is performed in reverse order.

Each island from 1 to 20 is equipped with a mechanism that operates as an EDA-type evolutionary algorithm (Fig. 1). Additionally, the islands are disaggregated according to the structure of the individuals in the population, which will henceforth be referred to as the stochastic sequence generator. Each island from 1 to 20 will have pop individuals (stochastic generators) with the same structure. In turn, these generators depend on how the parameters that define them are updated. Furthermore, an additional island is introduced, where a traditional genetic algorithm is implemented (island 21). All islands evolve synchronously, but structure prediction presents a computational bottleneck. Although each island independently generates candidate sequences, all sequences rely on the same instance of ESMFold for structure prediction, which is executed sequentially on a single GPU.

The individuals on the islands are constructed from six stochastic models (Supplementary Table 12). In model 1, for each position in the sequence, the probability of occurrence of each amino acid is determined. The individuals on islands 1, 2, 3 and 4 are constructed on the basis of this model. Models 2–4 include knowledge of amino acids—for example, how they are grouped on the basis of properties such as polarity, propensity to form part of a specific secondary structure or evolutionary similarity. In this way, three hierarchical models are constructed, where for each position in the sequence the probability of belonging to one of the groups is determined first, and then, given the current group, the probability of belonging to the next group is determined, and so on until the amino-acid level is reached. The individuals on islands 5–16 are constructed on the basis of these models. Model 5 is a Bayesian network where the nodes represent the positions in the sequence. In this network, an arc is drawn from each position to the next, reflecting the dependence between successive positions in the sequence. The individuals on islands 17 and 18 are constructed on the basis of model 5. Finally, model 6 represents a Markov chain between each pair of consecutive positions in the amino-acid sequence. In this model, the states correspond to the 20 amino acids and the individuals on islands 19 and 20 are constructed on the basis of this model.

Additionally, the update of the individuals’ parameters is carried out under four different schemes. In the first scheme (A, Supplementary Table 12), the parameters (probabilities) are calculated on the basis of the absolute frequencies of appearance obtained throughout the algorithm (islands 4, 8, 12, 16, 17 and 20). In the second scheme (B, Supplementary Table 12), the parameters (probabilities) are calculated from the absolute frequencies of appearance but resetting these every ngr generations (islands 3, 7, 11, 15, 18 and 19). In the third scheme (C, Supplementary Table 12), the parameters are calculated on the basis of the probability proportional to the size of the accumulated value of the function *d*_r∗_ throughout the algorithm (islands 2, 6, 10 and 14). In the fourth scheme (D, Supplementary Table 12), the parameters are calculated from the probability proportional to the size of the accumulated value of the function *d*_r∗_ but resetting this every ngr generations (islands 1, 5, 9 and 13), where ${d}_{\mathrm{r* }}=\max (1-\frac{d}{10},0)$ and *d* is the distance between the C_α_ atoms located at position r_*_ in the reference protein and the designed protein after superposition.

To update the parameters of the stochastic generator in generation *i*, two sets of sequences are selected. The first set contains m* sequences, while the second set contains t* sequences. The first set is formed from the m* sequences with the highest fitness among the *m* sequences generated during generation *i* (m* ≤ *m*). To construct the second set, t* sequences are selected from the repository that contains the *t* sequences with the highest fitness generated in each island (t* ≤ *t*). The selection of the t* sequences is performed with probabilities proportional to their fitness sizes (the details for the update rules of the stochastic generators are given in Supplementary Table 13 and Supplementary Section 2).

The implemented island model, where each island employs a distinct stochastic generator within an EDA framework, seeks to maximize both exploration and exploitation of the vast protein sequence space. The six stochastic models capture complementary biological and statistical properties, from simple position-wise amino-acid probabilities to hierarchical and context-aware models such as Bayesian networks and Markov chains. Combined with four diverse parameter update strategies, this design introduces a range of learning dynamics across islands. Such diversity improves the algorithm’s ability to escape local optima, enhances robustness across protein classes and enables the transfer of information through migration. A traditional genetic algorithm implemented on island 21 further complements this set-up, supporting recombination-driven exploration. This heterogeneous, multi-island architecture provides a flexible and scalable foundation for general protein sequence design.

### Proteins to design

To test the quality of the solutions produced by the KCM, we selected a small subset of proteins from the CATH Protein Structure Classification database v.4.3.0, which is a free and publicly available online resource that provides information on the evolutionary relationships of protein domains^61^ and classifies them according to the secondary structures that compose them. This database is available at http://download.cathdb.info/cath/releases/all-releases/v4_3_0/cath-classification-data/cath-domain-description-file-v4_3_0.txt.

In particular, we selected protein segments containing mainly α-helices and β-sheets. We also selected some proteins (Supplementary Table 14) that did not have well-defined secondary structures. In total, 23 proteins were selected, each between 15 and 33 residues long. We selected these proteins because they represent the three most frequent secondary structure types found in experimentally determined protein structures: α-helices, β-sheets and disordered regions. Additionally, we included test proteins that contain a combination of both α-helices and β-sheets.

### KCM installation

The KCM algorithm can be installed locally as a standalone Python program using the Anaconda package manager. Detailed installation instructions are available in the repository’s README file^62^. For improved performance, we recommend installing a local copy of the ESMFold model. To do this, first install ESMFold using the environment.yml file provided in its official GitHub repository^63^, which creates the esmfold environment. Once this is done, activate this environment using the conda activate esmfold command and follow the instructions described in listing 1.

Before running the KCM program, ensure that the esmfold environment is activated.

#### Listing 1

The Anaconda commands to install KCM’s required packages.

1. conda activate esmfold
2. conda install numpy=1.21
3. conda install scipy=1.7.1
4. conda install -c conda-forge biopython=1.79
5. conda install -c conda-forge pandas=1.3.5
6. conda install -c conda-forge matplotlib=3.5.3
7. conda install conda-forge::transformers=4.28.1
8. conda install pyrosetta=2020.10

### Peptide synthesis

All peptides for the experiments were obtained from Pepmic and synthesized using solid-phase peptide synthesis with the fluorenylmethyloxycarbonyl protecting group strategy. Peptides were purified using reverse-phase liquid chromatography (purity >95%).

### Bacterial strains and growth conditions

In this study, we used the following pathogenic bacterial strains: *A. baumannii* ATCC 19606, *E. coli* AIC221 (*E. coli* MG1655 phnE_2::FRT (control strain for AIC222)) and *E. coli* AIC222 (*E. coli* MG1655 pmrA53 phnE_2::FRT (polymyxin resistant; colistin-resistant strain)), *Klebsiella pneumoniae* ATCC 13883, *Pseudomonas aeruginosa* PAO1, *P. aeruginosa* PA14 and *Staphylococcus aureus* ATCC 12600. All pathogens were grown in Luria–Bertani (LB) broth and on LB agar. Pseudomonas isolation (*P. aeruginosa* strains) agar plates were exclusively used in the case of *Pseudomonas* species. In all the experiments, bacteria were inoculated from one isolated colony and grown overnight (16 h) in liquid medium at 37 °C. On the following day, inocula were diluted 1:100 in fresh media and incubated at 37 °C to mid-logarithmic phase.

### MIC assays

Broth microdilution assays^64^ were conducted to establish the MIC for each peptide. Peptides were added to untreated polystyrene 96-well microtiter plates and serially diluted twofold in sterile water, ranging from 1 to 64 μmol l^−1^. A bacterial inoculum at a concentration of 10^6^ CFU ml^−1^ in LB medium was then mixed in a 1:1 ratio with the peptide solution. The MIC was determined as the lowest peptide concentration that completely inhibited bacterial growth after 24 h of incubation at 37 °C. Each assay was performed in three independent replicates.

### Circular dichroism experiments

The circular dichroism experiments were conducted using a J1500 circular dichroism spectropolarimeter (JASCO) in the Biological Chemistry Resource Center at the University of Pennsylvania. Experiments were performed at 25 °C; the spectra graphed are an average of three accumulations obtained with a quartz cuvette with an optical path length of 1.0 mm, ranging from 260 to 190 nm at a rate of 50 nm min^−1^ and a bandwidth of 0.5 nm. The concentration of all peptides tested was 50 μmol l^−1^, and the measurements were performed in water, in a mixture of TFE and water with a 3:2 ratio, in a mixture of methanol and water with a 1:1 ratio and in SDS in water at 10 mmol l^−1^, with respective baselines recorded before measurement. A Fourier transform filter was applied to minimize background effects. Secondary-structure fraction values were calculated using the single-spectrum analysis tool on the server BeStSel^65^. Ternary plots were created in https://www.ternaryplot.com/ and subsequently edited.

### Outer-membrane permeabilization assays

NPN uptake assay was used to evaluate the ability of the peptides to permeabilize the bacterial outer membrane. Inocula of *A. baumannii* ATCC 19606 were grown to an optical density at 600 nm of 0.4 ml^−1^, centrifuged (9,391 *g* at 4 °C for 10 min), washed and resuspended in 5 mmol l^−1^ HEPES buffer (pH 7.4) containing 5 mmol l^−1^ glucose. The bacterial solution was added to a white 96-well plate (100 μl per well) together with 4 μl of NPN at 0.5 mmol l^−1^. Subsequently, peptides diluted in water were added to each well, and the fluorescence was measured at excitation wavelength (*λ*_ex_) = 350 nm and emission wavelength (*λ*_em_) = 420 nm over time for 45 min. The relative fluorescence was calculated using the untreated control (buffer + bacteria + fluorescent dye) as baseline and the following equation was applied to reflect the percentage difference between the baselines and the sample:

$$\begin{array}{l}{\mathrm{percentage}}\; {\mathrm{difference}}\\=\displaystyle\frac{100\times ({\mathrm{{{fluorescence}}_{{sample}}}}-{\mathrm{{{fluorescence}}_{\rm{untreated}\;\rm{control}}}})}{{\mathrm{{{fluorescence}}_{\rm{untreated}\;\rm{control}}}}}\end{array}.$$

### Cytoplasmic-membrane depolarization assays

The cytoplasmic-membrane depolarization assay was performed using the membrane-potential-sensitive dye DiSC_3_-5. *A. baumannii* ATCC 19606 in the mid-logarithmic phase were washed and resuspended at 0.05 optical density ml^−1^ (optical value at 600 nm) in HEPES buffer (pH 7.2) containing 20 mmol l^−1^ glucose and 0.1 mol l^−1^ KCl. DiSC_3_-5 at 20 μmol l^−1^ was added to the bacterial suspension (100 μl per well) for 15 min to stabilize the fluorescence, which indicates the incorporation of the dye into the bacterial membrane, and then the peptides were mixed 1:1 with the bacteria to a final concentration corresponding to their MIC values. Membrane depolarization was then followed by reading changes in the fluorescence (*λ*_ex_ = 622 nm, *λ*_em_ = 670 nm) over time for 60 min. The relative fluorescence was calculated using the untreated control (buffer + bacteria + fluorescent dye) as baseline and the following equation was applied to reflect the percentage difference between the baselines and the sample:

$$\begin{array}{l}{\mathrm{percentage}}\; {\mathrm{difference}}\\=\displaystyle\frac{100\times ({\mathrm{{{fluorescence}}_{\rm{sample}}}}-{\mathrm{{{fluorescence}}_{\rm{untreated}\; \rm{control}}}})}{{\mathrm{{{fluorescence}}_{\rm{untreated}\; \rm{control}}}}}\end{array}.$$

### Eukaryotic cell culture conditions

HEK293T cells were obtained from the ATCC (CRL-3216). The cells were cultured in high-glucose DMEM supplemented with 1% penicillin and streptomycin (antibiotics) and 10% fetal bovine serum and grown at 37 °C in a humidified atmosphere containing 5% CO_2_.

### Cytotoxicity assays

One day before the experiment, an aliquot of 100 μl of the cells at 50,000 cells ml^−1^ was seeded into each well of the cell-treated 96-well plates used in the experiment (that is, 5,000 cells per well)^45^. The attached HEK293T cells were then exposed to increasing concentrations of the peptides (4–64 μmol l^−1^) for 24 h. After the incubation period, we performed the (3-(4,5-dimethylthiazol-2-yl)-2,5-diphenyltetrazolium bromide) tetrazolium reduction assay (MTT assay)^45^. The MTT reagent was dissolved at 0.5 mg ml^−1^ in medium without phenol red and was used to replace cell culture supernatants containing the peptides (100 μl per well), and the samples were incubated for 4 h at 37 °C in a humidified atmosphere containing 5% CO_2_, yielding the insoluble formazan salt. The resulting salts were then resuspended in hydrochloric acid (0.04 mol l^−1^) in anhydrous isopropanol and quantified by spectrophotometric measurements of absorbance at 570 nm. All assays were carried out as three biological replicates.

### Skin abscess infection mouse model

The backs of six-week-old female CD-1 mice under anaesthesia were shaved and injured with a superficial linear skin abrasion made with a needle. An aliquot of *A. baumannii* ATCC 19606 (2 × 10^6^ CFU ml^−1^; 20 μl) previously grown in LB medium until 0.5 optical density ml^−1^ (optical value at 600 nm) and then washed twice with sterile PBS (pH 7.4, 9,391 *g* for 3 min) was added to the scratched area. Peptides diluted in sterile water at MIC value were administered to the wound area 1 h after the infection. Two and four days after infection animals were euthanized, and the scarified skin was excised, homogenized using a bead beater (25 Hz for 20 min), tenfold serially diluted, and plated on McConkey agar plates for CFU quantification. The experiments were performed using six mice per group. Mice were housed in groups of three and maintained under a 12-h light/dark cycle at 22 °C with humidity controlled at 50%. The skin abscess infection mouse model was revised and approved by University Laboratory Animal Resources from the University of Pennsylvania (protocol 806763).

### Deep thigh infection mouse model

Six-week-old female CD-1 mice were rendered neutropenic by two doses of cyclophosphamide (150 mg kg^−1^ and 100 mg kg^−1^) applied intraperitoneally 3 and 1 d before the infection. On day 4 of the experiment, the mice were infected in their right thighs with a 100-μl intramuscular injection of the *A. baumannii* ATCC 19606 in PBS at concentration of 5 × 10^6^ CFU ml^−1^. The bacterial cells were grown in LB broth, washed twice with PBS solution and diluted at the desired concentration. The peptides were administrated intraperitoneally 2 h after the infection. Two days after infection mice were euthanized, and the tissue from the right thigh was excised, homogenized using a bead beater (25 Hz for 20 min), tenfold serially diluted, and plated on McConkey agar plates for bacterial colony counting. The experiments were performed using six mice per group. Mice were housed in groups of three and maintained under a 12-h light/dark cycle at 22 °C with humidity controlled at 50%. The deep thigh infection mouse model was revised and approved by University Laboratory Animal Resources from the University of Pennsylvania (protocol 807055).

### Reporting summary

Further information on research design is available in the Nature Portfolio Reporting Summary linked to this article.

## Supplementary information

Supplementary Information Supplementary Notes, Supplementary Tables S1 – S14, and Supplementary Figures S1 – S6.

Reporting Summary

Supplementary Data 1 Statistical data for the ternary plots of the comparison between our method and ESM-IF1, ProteinSolver and ProteinMPNN.

Supplementary Data 2 Statistical data for the backbone superposition of the designed α/β-proteins with their references.

Supplementary Data 3 Statistical data for the runtime of ESMFold as a function of sequence length.

Supplementary Data 4 Statistical data for the circular dichroism spectra and heatmap with the percentage of secondary structure obtained on the BeStSel server.

Supplementary Data 5 Statistical data for the outer-membrane permeabilization and cytoplasmic-membrane depolarization fluorescence measurements.

Supplementary Data 6 Statistical data for the cytotoxicity experiments: cell viability versus peptide concentration, anti-infective activity in deep thigh infection and mouse weight changes over time in both skin scarification and deep thigh infection mouse models.

## Source data

Source Data Fig. 1 Zip containing the pdb files for each target and the corresponding best design.

Source Data Fig. 1 Source data for the RMSD_S and GDT_TS for the 100 best designs for each target peptide, GDT_TS as a function of RMSD_S of the highest fitness design for each protein and superposition of target and designed peptides.

Source Data Fig. 2 Source data for the minimal inhibitory concentration heatmap and circular dichroism ternary plots.

Source Data Fig. 3 Source data for the outer membrane permeabilization and cytoplasmic membrane depolarization relative fluorescence measurements and cytotoxic concentration needed to kill 50% of mammalian cells (CC_50_) heatmap.

Source Data Fig. 4 Source data for the violin plots of the anti-infective activity of the peptides in vivo.

## Data Availability

The test data for in silico experiments are available at GitHub (https://github.com/LabBiocomp/KCM; ref. ^65^). All data pertaining to the experimental validation of generated peptides are available in the Supplementary Data or from the corresponding authors upon reasonable request. Source data are provided with this paper.

## Code availability

The KCM algorithm is available at GitHub (https://github.com/LabBiocomp/KCM; ref. ^62^) and Zenodo (10.5281/zenodo.16921598; ref. ^66^).
