# Supplementary material for: Tailored structured peptide design with a key-cutting machine approach
Source: Nat Mach Intell. 2025 Oct 21;7(10):1685–97. doi: 10.1038/s42256-025-01119-2 (PMC12552119; doi:10.1038/s42256-025-01119-2)
Supplement: Supplementary file 1 — Supplementary Notes, Supplementary Tables S1 – S14, and Supplementary Figures S1 – S6. [file 42256_2025_1119_MOESM1_ESM.pdf]

nature machine intelligence

Supplementary information https://doi.org/10.1038/s42256-025-01119-2

Tailored structured peptide design with a key-cutting machine approach

In the format provided by the authors and unedited

Table of Contents

Similarity Metrics 2
Stochastic generator of sequences 5
Table S1. Parameter values of KCM used in the design of selected proteins 8
Table S2. Statistics of the 100 best designs for each protein 9
Table S3. Bayesian Test with 50 solutions 9
Table S4. Bayesian Test wih 250 solutions 10
Table S5. Proteins with mixed α-helices and β-sheets secondary structures, selected for design using the KCM approach 10
Table S6. The 100 best designs for each αβ protein structure 10
Table S7. Parameter values used in KCM to design IDR 2009 11
Table S8. Ten best designs per combination of objective function terms 12
Table S9. Selected peptides for experimental validation 12
Table S10. Similarity criteria for the synthesized peptides using the backbone of IDR 2009 as a template 13
Table S11. Description of the selected iLearn descriptors 14
Table S12. Combination of models and parameters update strategies used in every island 15
Table S13. The parameters associated with models 15
Table S14. Proteins selected for design using our algorithm 16
Figure S1. Sign test and Bayesian paradigm, comparison between KCM, ESM-IF1, Protein Solver, and ProteinMPNN 17
Figure S2. Backbone superposition of the designed α/β-proteins with their references 19
Figure S3. Box plot showing the runtime of ESMFold as a function of sequence length 19
Figure S4. Circular dichroism spectra of designed peptides 20
Figure S5. Outer membrane permeabilization and cytoplasmic membrane depolarization of *A. baumannii* ATCC 19606 induced by designed peptides 21
Figure S6. Cytotoxicity and anti-infective activity of designed peptides 22

S1. Similarity metrics

Notation

Let A_m and A_r denote respectively the set of atoms being considered in the model (designed) structure and the reference (or target) structure. Unless otherwise stated, we only consider the $C\alpha$ atoms from each residue in the protein backbone. The number of atoms in a given structure is denoted as $n \in N$, where $n = |A_m| = |A_r|$. We will frequently use $a_{m,i} \in A_m$ and $a_{r,i} \in A_r$ to denote the ith pair of corresponding atoms in the model and reference structures. Furthermore, let $a, b \in A_m \cup A_r$ be the Cartesian coordinates of two atoms, we denote the Euclidean distance between these atoms as $d(a, b)$. We will also use $D_i = d(a_{m,i}, a_{r,i})$ as a shorthand.

S1.1. RMSD_S

The RMSD measures the average distance the atoms of the model structure deviate from their corresponding position in the reference structure[48,49]. The RMSD_S is measured in Angstroms and is computed after superimposing the two structures being compared. The RMSD_S is a positive real number, a value of zero indicates that the two structures are identical. The formula for calculating the RMSD_S is given by the following equation:

$$RMSD\_S = \sqrt{\frac{1}{n}\sum_{i=1}^{n} D_i^2}$$

For calculating the RMSD_S value, we will consider not only the C_α atoms, but also the N, C and O atoms from each residue in the backbone.

S1.2. TM score

Like the RMSD, the TM Score also measures the average deviation distance between corresponding atoms in the model and reference structures after superimposing said structures. However, in the TM Score, the deviation distances are normalized by the number of atoms[50]. This allows the TM Score to describe more consistently the similarity between two structures regardless of their size. Also, since large deviation distances end up having a smaller contribution to the total score than short deviations, the TM Score is less sensitive than the RMSD to local misalignments in the structures. The TM Score is a real number in (0,1], where 1 indicates that the two structures are identical. The TM Score is given by the following formula:

$$TM\_S = \frac{1}{n}\sum_{i=1}^{n} \frac{1}{1 + \left[\frac{D_i}{d_0(n)}\right]^2}$$

where $d_0(n)$ is the scaling factor determined by the size of the protein. This scaling factor is computed as:

$$d_0(n) = 1.24(n - 15)^{1/3} - 1.8$$

S1.3. GDT_TS

This score can be interpreted as the percentage of atoms in the model structure which deviate from their corresponding position in the reference structure in a distance that is within a given tolerance threshold (called cutoff distance)[33]. Usually, multiple cutoff distances are used, and the GDT_TS is the average of the percentages computed for each of these distances. The calculation of GDT_TS requires that the model and reference structures be superimposed. Being a percentage,

the GDT_TS score is a real value in the interval [0,100], where 100 indicates that the two structures are identical under the smallest distance threshold.

Let S be the set of cutoff distances, and let $k = |S|$. Usually, the cutoff distances are $S = \{1,2,4,8\}$[33]. For all cutoff distances $s \in S$, let $g_s: N \rightarrow \{0,1\}$ be a function defined as follows:

$$g_s(i) = \begin{Bmatrix} 1 \ if \ D_i \leq s \\ 0 \ if \ D_i > s \end{Bmatrix}$$

where $1 \leq i \leq n$. Then, the GDT_TS is given by:

$$GDT\_TS = \frac{1}{nk} \sum_{s \in S} \sum_{i=1}^{n} g_s(i)$$

S1.4. RMSD_DM

The contact map of a protein refers to an alternative representation of the structure in which the distance between every pair of protein residues is described[58]. The contact map is a bidimensional symmetric matrix with binary values in which an entry value of 1 indicates that the distance between the corresponding residues is larger than a given contact threshold, and a value of zero indicates otherwise[59]. The same concept applies to the distance matrix, except that the Euclidean distances between each pair of atoms are directly stored as the matrix's entries.

Let A be the set of atoms defining some protein structure. The distance matrix of A, denoted as M_A, is the $n \times n$ matrix defined as:

$$M_A = \begin{bmatrix} m_{11}^A & m_{12}^A & \cdots m_{1n}^A \\ m_{21}^A & m_{22}^A & \cdots m_{2n}^A \\ \cdots & \cdots & \cdots \ \cdots \\ m_{n1}^A & m_{n2}^A & \ldots m_{nn}^A \end{bmatrix} = \begin{bmatrix} d(a_1, a_1) & d(a_1, a_2) & \cdots \ d(a_1, a_n) \\ d(a_2, a_1) & d(a_2, a_2) & \cdots \ d(a_2, a_n) \\ \cdots & \cdots & \cdots \ \cdots \\ d(a_n, a_1) & d(a_{n1}, a_2) & \ldots d(a_n, a_n) \end{bmatrix} \quad (10)$$

where, for all $1 \leq i \leq n$, the symbol $a_i \in A$, denotes the ith atom in the structure A.

The contact map can then be constructed from the distance map. Let $\varepsilon \in R$ be the contact threshold, and let $h_\varepsilon: R \rightarrow \{0,1\}$ be a function defined as:

$$h_\varepsilon(x) = \begin{Bmatrix} 1 \ if \ x \leq \varepsilon \\ 0 \ if \ x > \varepsilon \end{Bmatrix} \quad (11)$$

Then, the contact map of A, denoted as C_A, is the $n \times n$ matrix defined as:

$$C_A = \begin{bmatrix} c_{11}^A & c_{12}^A & \cdots c_{1n}^A \\ c_{21}^A & c_{22}^A & \cdots c_{2n}^A \\ \cdots & \cdots & \cdots \ \cdots \\ c_{n1}^A & c_{n2}^A & \ldots c_{nn}^A \end{bmatrix} = \begin{bmatrix} h_\varepsilon(m_{11}^A) & h_\varepsilon(m_{12}^A) & \cdots h_\varepsilon(m_{1n}^A) \\ h_\varepsilon(m_{21}^A) & h_\varepsilon(m_{22}^A) & \cdots h_\varepsilon(m_{2n}^A) \\ \cdots & \cdots & \cdots \ \cdots \\ h_\varepsilon(m_{n1}^A) & h_\varepsilon(m_{n2}^A) & \ldots h_\varepsilon(m_{nn}^A) \end{bmatrix} \quad (12)$$

Given the distance maps from the model and reference structures, denoted M_{A_m} and M_{A_r} respectively, the RMSD_DM score is computed using the following formula:

$$RMSD\_DM = \sqrt{\frac{2}{n(n-1)} \sum_{i=1}^{n-1} \sum_{j=i+1}^{n} \left(m_{i,j}^{A_m} - m_{i,j}^{A_r}\right)^2} \quad (13)$$

S1.5. ESM-2 descriptors

ESM-2 is a family of large Transformer-based neural networks that learned global protein properties and inter-residual relationships from known amino acid sequences[52,60]. These models were trained using a masking method, where random letters in the sequence were obscured and the neural network had to correctly predict them[61]. These models have proven to be useful for both computational protein design and protein structure prediction[62]. ESM-2 models are of particular interest in protein design because they can be used to produce proteins that do not belong to the collection of known proteins[63].

The ESM-2 model family consists of six pre-trained models, each with 6, 12, 30, 33, 36, and 48 layers[52,64]. All models were trained with 65 million unique sequences. Given an amino acid sequence, any ESM-2 model returns as output a real-valued vector for each residue in the sequence. The protein and per-residue properties and the inter-residual relationships learned by the model is encoded in this vector of descriptors[52,64]. Depending on the number of layers used, each model produces larger or smaller descriptor vectors. For example, the 33-layer model produces descriptor vectors with 1,280 values per residue, while the 48-layer model produces 5,120 values per residue.

S1.6. iLearn descriptors

iLearn is a software toolkit that integrates several algorithms and procedures for calculating, extracting, analyzing and selecting protein and nucleotide features[65]. A subset of nine of these feature calculators (mainly those describing physicochemical characteristics of the amino acid sequence) was selected to be utilized in our KCM (**Table S11**).

S1.7. Similarity between descriptors

Given the descriptor vectors for a model`s sequence and the reference`s sequence, the similarity between them can be computed by using the Kullback-Leibler divergence and the Jeffrey's distance[53]. Given two probability distributions, P and Q defined over the same set of data points X, the Kullback-Leibler divergence, denoted $D_{KL}(P||Q)$, measures how much distribution P diverges from the reference distribution Q. On the other hand, Jeffrey's distance, denoted D_J is simply a symmetric version of the Kullback-Leibler divergence[53]. In the context of our protein design algorithm, distribution P would be obtained from the iLearn and ESM-2 descriptor vectors of a model sequence, and distribution Q would be obtained from the iLearn and ESM-2 descriptor vectors of the reference sequence.

Kullback-Leibler divergence is computed as[53]:

$$D_{KL}(P||Q) = \sum_{x \in X} P(x)\ ln\ ln\ \frac{P(x)}{Q(x)} \tag{14}$$

Meanwhile, Jeffrey's distance is simply computed as[53]:

$$D_J(Q,P) = D_J(P,Q) = D_{KL}(P||Q) + D_{KL}(Q||P) \tag{15}$$

All the selected iLearn feature calculators produce real-valued descriptor vectors (**Table S11)**. Some of these vectors (like the ones produced by GDPC and GTPC) contain relative frequency values in $[0,1]$, while others (like the ESM-2 descriptor vectors) contain absolute values, absolute frequency or percentage point values. In the first case, the relative frequency values are used directly as the probability distribution P or Q for calculating Jeffrey's distance. In the second case, a histogram is computed, dividing the descriptor values' range into 20 equal-sized subintervals and counting the number of values in each subinterval. These numbers are then converted into relative frequency values that are used as the distribution P or Q for calculating Jeffrey's distance.

S2. Stochastic generator of sequences

The stochastic sequence generator is based on six mathematical models designed to learn how the highest fitness sequences are distributed within the feasible solution space. The generator also depends on the procedure used to update the parameters defined in the models. The first four are hierarchical models that learn the distribution of amino acids at each position in the sequence, without considering the relationships between different positions. To address these relationships, two models are employed: a Bayesian network and a Markov chain, which account for the interactions between different positions in the sequence.

The hierarchical models classify amino acids into different groups according to certain properties that they share with others. These properties are polarity, propensity to forming a certain secondary structure[66,67], and structural and evolutionary similarity[65,68]. The latter refers to the fact that the substitution of one amino acid for another has a higher probability of being accepted if the two residues belong to the same group. The amino acids classification into different groups is as follows. The groups according to their polarity are:

- Hydrophilic: $C_{11} = \{K, R, H, N, E, D, Q\}$
- Hydrophobic: $C_{12} = \{F, I, M, V, L\}$
- Moderate polarity: $C_{13} = \{Y, W, T, S, P, A, G, C\}$

The groups according to the propensity of forming different secondary structures are:

- α-helices: $C_{21} = \{L, A, Q, M, E, R, K\}$
- β-sheet: $C_{22} = \{S, N, D, P, G\}$
- Loops: $C_{23} = \{Y, T, W, F, I, V\}$
- Others: $C_{24} = \{H, C\}$

The groups according to their evolutionary relationship are:

- $C_{31} = \{Y, F, W\}$
- $C_{32} = \{R, K, H\}$
- $C_{33} = \{D, Q, N, E\}$
- $C_{34} = \{V, L, I, M\}$
- $C_{35} = \{S, G, P, T, A\}$
- $C_{36} = \{C\}$

Lastly, the groups according to their structural similarity are:

- $C_{41} = \{D, E\}$, $C_{42} = \{N, Q\}$, $C_{43} = \{V, L\}$, $C_{44} = \{S, T\}$,
- $C_{45} = \{A, G\}$, $C_{46} = \{K\}$, $C_{47} = \{R\}$, $C_{48} = \{H\}$,
  $C_{49} = \{I\}$, $C_{4,10} = \{M\}$, $C_{4,11} = \{F\}$, $C_{4,12} = \{Y\}$,
- $C_{4,13} = \{W\}$, $C_{4,14} = \{P\}$, $C_{4,15} = \{C\}$

Model 1 is the more general model and does not assume any previous knowledge about the amino acids or how they can be grouped together. Thus, for each position in the sequence, only the probability that a certain amino acid occupies that position is determined in this model. Models 2, 3, and 4 must determine the probability that a given amino acid belongs to one of such groups, and then, given a certain group, determine the probability of belonging to the next group. The order in which the different groups are visited is as follows:

- Model 2: $C_{1i} \rightarrow C_{3j} \rightarrow C_{4k} \rightarrow a_l$
- Model 3: $C_{1i} \rightarrow C_{2o} \rightarrow C_{3j} \rightarrow a_l$
- Model 4: $C_{2o} \rightarrow C_{1i} \rightarrow C_{3j} \rightarrow a_l$

where a_l denotes each of the 20 standard amino acids, and $l = \underline{1,...,20}$, $i = \underline{1,...,3}$, $j = \underline{1,...,6}$, $k = \underline{1,...,15}$, $o = \underline{1,...,4}$. **Table S13** lists the parameters to be estimated by each model, 1 through 4.

In a Bayesian network, the number of parameters to be estimated increases with the number of arcs that are defined and, consequently, the number of samples needed to determine the parameters is higher. Thus, a Bayesian network is defined as follows: each position of the sequence corresponds to a node of the network, an arc is established between two nodes if they are in consecutive positions and the arc will point towards the node that is in a higher position. Model 5 will be a Bayesian network and the parameters it must estimate are listed in **Table S13**.

Matrix P^q can be thought of as the transition array at time 1 of a Markov chain. Also, given the nature of such transition matrix, the Markov chain would have a stationary distribution, and, consequently, the transition matrix can be determined when the time tends to infinity $P^{q\infty}$. In this way, $P^{q\infty}$ is used to estimate the amino acids in the sequence. Model 6 will be a Markov chain that needs to estimate the parameters shown in **Table S13**.

The stochastic sequence generator depends on the method used to estimate and update the parameters in each generation. Therefore, for each parameter θ_k^{ijr} in models 1 to 4, with fixed i, j and *r*, we have the arrays A^{ijr} (type A) or B^{ijr} (type B), depending on the approach used. Both arrays initially have value 1 in their positions and, depending on the case, the probability assigned to each parameter is proportional to its corresponding value stored in the arrays A^{ijr} and B^{ijr}.

The arrays A^{ijr} or B^{ijr} are updated at the end of each generation, and the parameters are then recalculated. The process of updating the parameters will be explained with an example: assume that, on a given island, the model *m=4* is being used to learn how the amino acids are distributed at each position of the sequence. Also assume that, at position $r*$ of the sequence number n_1 selected to be updated, there is amino acid a_{l*} and $a_{l*} \in C_{3j*}, a_{l*} \in C_{1i*}$ and $a_{l*} \in C_{2o*}$, where $l^* \in \underline{1,...,20}$, $i^* \in \underline{1,...,3}$, $j^* \in \underline{1,...,6}$, $o^* \in \underline{1,...,4}$ and $r^* \in \underline{1,...,n}$. Under these conditions, the arrays $\{A, B\}^{ijr}$ are updated as follows:

$$\{A, B\}_{l*}^{m4r*} = \{A, B\}_{l*}^{m4r*} + \{1, d_{n1,r*}\},$$

$$\{A, B\}_{j*}^{m3r*} = \{A, B\}_{j*}^{m3r*} + \{1, d_{n1,r*}\},$$

$$\{A, B\}_{i*}^{m2r*} = \{A, B\}_{i*}^{m2r*} + \{1, d_{n1,r*}\},$$

$$\{A, B\}_{o*}^{m1r*} = \{A, B\}_{o*}^{m1r*} + \{1, d_{n1,r*}\},$$

where $d_{n1,r*} = max(1 - \frac{d}{10}, 0)$ and d is the distance between the C_α atoms of the corresponding amino acid in the reference sequence after both proteins are superimposed.

In this way, updating the arrays $\{A, B\}^{ijr}$ is performed for the amino acids corresponding to all the positions of all the sequences selected for updating their parameters. The probabilities of each parameter are then calculated proportionally to the values stored in the arrays $\{A, B\}^{ijr}$. Additionally, the information stored in type A arrays corresponds to the frequency of appearance of amino acids or groups in the positions. Meanwhile, the information stored in type B is more selective, since, if the amino acid appears in that position but after superposition of the C_α atoms

are distanced more than 10 Å away, then the information stored regarding that amino acid or group is negligible.

Due to the nature of models 5 and 6, type A arrays are always used to update their associated parameters. That is, the stored information corresponds to the absolute frequencies of the amino acids. In this way, all the tools are already given to explain how the islands are disaggregated according to the stochastic generator and the way the generator is used to update and calculate the parameters.

Four islands were built for each of the models 1 to 4. Two of these islands use type A arrays to determine the parameters, while the other two use type B arrays. Likewise, the two islands that use the same type of array differ in that the corresponding values in each array in one of the models are incremented throughout the algorithm, while in the other model, the values are reset every ngr generations. For models 5 and 6, only two islands were built, both of which use type A arrays. As in the previous case, these islands are different in that the values stored in those arrays are reset every ngr generations. In total, 20 islands were built that feature the stochastic sequence generator, while island 21 implements a traditional genetic algorithm explained above.

Table S1. Parameter values of KCM used in the design of selected proteins. *ng*: Maximum number of generations; *gdt*: GDT_TS threshold used as a stopping criterion; *s*: Number of islands; *p*: Information exchange rate between islands; *pop*: Population size, *t*: Number of elite sequences stored on each island; m_l: Number of sequences selected for migration between islands; *m*: Number of sequences sampled at each generation; *ngr*: Number of generations after which parameters are reset for islands 1, 3, 5, 7, 9, 11, 13, 15, 17, 18, and 19; *m**: Number of best sequences (from the m samples) used for parameter updates; *t**: Number of best sequences (from the t stored sequences on each island) used for parameter updates.

Parameter	Value
Ng	Dependent of the protein
Gdt	0.9 for β-sheets and 1 for the rest
P	0.1
Pop	5
T	150
S	All 21 islands
M	5
Ml	1
Ngr	10
m*	3
t*	2

Table S2. Statistics of the 100 best designs for each protein. 2D: is the corresponding secondary structure, AH: alpha helix, BS: beta sheets, ND: not defined, L: character corresponding to each protein in Figure 3, PDB id.: the four letter protein ID in the PDB, RMSD_S Min, Mean and Sd: minimum, mean value and standard deviation for RMSD_S of the 100 designs of best fitness for each protein, GDT_TS Max, Mean and Sd: maximum, mean value and standard deviation for GDT_TS corresponding to the 100 best fitness for each protein, Identity: is the identity percentage of the highest fitness design with the reference sequence, n: length of the selected subsequence from the protein, and *ng*: number of generations employed in the design.

		**RMSD_S**			**GDT_TS**					
**2D**	**L**	**Min**	**Mean**	**Sd**	**Max**	**Mean**	**Sd**	**Identity (%)**	**N**	**ng**
BS	A	1.45	2.01	0.21	0.79	0.73	0.03	11	27	1,000
BS	B	1.19	2.05	0.40	0.91	0.73	0.07	12	26	210
BS	C	2.05	2.52	0.22	0.75	0.68	0.03	4	27	1,000
BS	D	1.10	2.00	0.38	0.92	0.74	0.05	12	26	305
BS	E	1.02	1.89	0.19	0.91	0.74	0.04	17	23	250
BS	F	1.61	2.17	0.23	0.84	0.70	0.04	10	30	1,000
BS	G	1.11	1.49	0.14	0.90	0.82	0.03	7	28	890
BS	H	2.27	2.64	0.22	0.72	0.66	0.03	3	33	1,000
AH	I	0.80	1.56	0.36	0.97	0.86	0.06	11	19	100
AH	J	0.36	0.46	0.06	1.0	0.99	0.01	9	22	100
AH	K	0.40	0.66	0.11	1.00	0.98	0.02	20	20	100
AH	L	2.19	2.82	0.29	0.70	0.59	0.04	6	19	100
AH	M	0.28	0.41	0.04	1.00	0.99	0.00	5	17	100
AH	N	0.47	0.74	0.13	0.99	0.97	0.01	20	20	100
AH	O	0.48	0.58	0.06	0.99	0.99	0.00	12	17	100
AH	P	0.47	0.61	0.07	1.00	0.98	0.01	0	19	100
AH	Q	0.41	0.69	0.13	1.00	0.97	0.02	16	19	100
AH	R	0.30	0.38	0.05	1.00	0.99	0.00	0	17	100
AH	S	0.54	0.85	0.19	0.99	0.95	0.02	24	17	100
AH	T	0.88	1.19	0.14	0.95	0.89	0.03	17	18	100
ND	U	2.01	2.58	0.22	0.70	0.61	0.04	13	15	100
ND	V	2.01	2.83	0.33	0.70	0.59	0.05	0	15	100
ND	W	3.20	4.15	0.49	0.55	0.47	0.03	0	28	100

Table S3. **Bayesian Test with 50 solutions**. Probability of belonging to each region, resulting from the sign test (Left, Upper, and Right). Best 50 solutions. Left is the probability of KCM losing against the other methods, Upper is the probability of equal performance, and Right is the probability of KCM wining.

**Region**	**ESM-IF1**		**Protein Solver**		**ProteinMPNN**	
	GDT_TS	RMSD_S	GDT_TS	RMSD_S	GDT_TS	RMSD_S
Left	0.3158	0.49245	0.00	0.00	0.17835	0.404
Upper	0.4892	0.0126	0.0045	0.0001	0.64405	0.00315
Right	0.195	0.49495	0.9955	0.9999	0.1776	0.59285

Table S4. **Bayesian Test wih 250 solutions.** Probability of belonging to each region, resulting from the sign test (Left, Upper, and Right). Best 250 solutions. Left is the probability of KCM losing against the other methods, Upper is the probability of equal performance, and Right is the probability of KCM wining.

**Region**	**ESM-IF1**		**Protein Solver**		**ProteinMPNN**	
	GDT_TS	RMSD_S	GDT_TS	RMSD_S	GDT_TS	RMSD_S
Left	0.3183	0.3176	0	0	0.08085	0.3203
Upper	0.48485	0.01245	0.0051	0	0.7694	0.0131
Right	0.19685	0.66995	0.9949	1	0.14975	0.6666

Table S5. Proteins with mixed α-helices and β-sheets secondary structures, selected for design using the KCM approach. CATH ID: seven-letter code of the protein in the CATH database; Sequence: the subsequence corresponding to the selected structure; n: length of the selected sequence; Protein name: the name of the protein in the PDB; 2D: secondary structure, where ab means alpha-beta.

**CATH ID**	**Sequence**	**n**	**Protein name (from PDB)**	**2D**
1AAYA02	KPFQCRICMRNFSRSDHLTTHIRTHTG	27	ZIF268 zinc Finger-DNA complex	ab
1F2IK01	RPYACPVESCDRRFSRSDELTRHIRIHTG	29	Cocrystal structure of selected zinc finger dimer bound to DNA	ab
1LLMC01	MKPFQCRICMRNFSRSDHLTTHIRTHTGEK	30	Crystal structure of ZIF23-GCN4 chimera bound to DNA	ab
2I13A05	KPYKCPECGKSFSRRDALNVHQRTH	25	Aart, a six finger zinc finger designed to recognize ANN triplets	ab
2PRTA03	VKPFQCKTCQRKFSRSDHLKTHTRTHTG	28	Structure of the Wilms tumor suppressor protein zinc finger domain bound to DNA	ab
3E7RL01	DDMQCHNHCKSIKGYKGGYCAKGGFVCKCY	30	X-ray crystal structure of Racemic Plectasin	ab
3VDUA02	LRYCKICFNITDKEVCDICSDEN	23	Structure of recombination mediator protein RecRK21G mutant	ab
4GZNC01	ERPFFCNFCGKTYRDASGLSRHRRAHLG	28	Mouse ZFP57 zinc fingers in complex with methylated DNA	ab

Table S6. The 100 best designs for each αβ protein structure. 2D: is the corresponding secondary structure, where AB means alpha-beta, BS: beta sheets, ID: the seven-letter ID in the CATH database, RMSD_S Min, Mean and Sd: minimum, mean value and standard deviation for RMSD_S of the 100 designs of best fitness for each protein, GDT_TS Max, Mean and Sd: maximum, mean value and standard deviation for GDT_TS corresponding to the 100 best fitness for each protein, Identity: is the identity percentage of the highest fitness design with the reference sequence, n: length of the selected subsequence from the protein, and *ng*: number of generations employed in the design.

		RMSD_S			GDT_TS					
**2D**	**ID**	**Min**	**Mean**	**Sd**	**Max**	**Mean**	**Sd**	**Identity (%)**	**N**	**ng**
AB	1AAYA02	1.40	1.89	0.18	0.91	0.81	0.03	12	27	350
AB	1F2IK01	1.73	2.09	0.17	0.79	0.73	0.03	3	29	350
AB	1LLMC01	2.10	2.71	0.26	0.73	0.69	0.03	14	30	350
AB	2I13A05	1.37	2.11	0.42	0.91	0.75	0.06	13	25	119
AB	2PRTA03	0.97	1.63	0.18	0.93	0.82	0.03	11	28	267
AB	3E7RL01	1.37	1.84	0.18	0.91	0.80	0.03	23	30	188
AB	3VDUA02	1.03	1.59	0.22	0.91	0.83	0.04	22	23	227
AB	4GZNC01	1.05	1.67	0.26	0.91	0.80	0.05	11	28	277

Table S7. Parameter values used in KCM to design IDR 2009.

Parameter	Value
ng	(A)-1770, (B)-1160, (C)-1080, (D)-470
gdt	1
P	0.10
pop	5
T	80
S	all the islands except island 21
M	3
m1	1
ngr	8
m*	2
t*	1

Table S8. Ten best designs per combination of objective function terms (target IDR-2009).

Model	Sequence
**AT**	HWKAAFVHWAIA, HWHAILIHFHFA, HWKAAFVHFAIA, AFLAIVLHFHIA HWHNKVLHWHIQ, WHLAAVLHWHIH, HWHACVVHIMIH, FRLAAVLHFHIH YFHAAVLHFAIH, IWHAAVLHFAIS
**NE**	PHAMAWLCIRFH, FKIHVWLCIIAF, KWKARIQFKAAH, WHFHVWIHCIAF EKWHQFLVVAFF, LQACHVHWFFFF, FKLCFVLHFVAL, YKLVFILCYHMH AFAALFWWHKIM, HLWCDWHQHILA
**NG**	HWKLIVRWKVQH, HWHLILHFHACH, HWHVIVHFHACH, RFKLCRIIVKYH KFKLIVHYRICK, KWKLIIRWRICH, HWHLIVHFHACH, RWKLIIRWRICH RWKLCRIIVKYH, KYKLILRFHICK
**ND**	IAANALAAGIFF, ALAAIVIGFIIA, FAAACVWGFIAA, ATAGVLVAFIIA AAAACLVAFIMA, ALAAIVIAIIMA, ALAAAWIAIIFD, IAAAIIAAIIMA LAAACVAGIIFA, ALAAILVAIIIN

Table S9. Selected peptides for experimental validation.

Name	Sequence
**AT1**	HWHAILIHFHFA
**AT2**	AFLAIVLHFHIA
**AT3**	HWHNKVLHWHIQ
**AT4**	FRLAAVLHFHIH
**AT5**	IWHAAVLHFAIS
**NE1**	PHAMAWLCIRFH
**NE2**	KWKARIQFKAAH
**NE3**	WHFHVWIHCIAF
**NE4**	FKLCFVLHFVAL
**NG1**	HWKLIVRWKVQH
**NG2**	HWHLILHFHACH
**NG3**	KFKLIVHYRICK

Table S10. Similarity criteria for the synthesized peptides using the backbone of IDR 2009 as a template.

ID	Energy	RMSD_S	GDT_TS	Descriptors similarity	Identity (%)
AT1	29.68	0.36	1.00	0.89	8
AT2	24.07	0.27	1.00	0.84	0
AT3	120.88	0.30	1.00	0.89	8
AT4	33.75	0.29	0.99	0.87	0
AT5	19.85	0.29	0.99	0.82	8
NE1	55.04	0.28	0.99	0.83	0
NE2	79.29	0.69	0.98	0.87	25
NE3	43.50	0.39	1.00	0.83	8
NE4	61.95	0.35	1.00	0.81	0
NG1	44.00	5.54	0.34	0.98	42
NG2	38.07	0.65	0.97	0.97	17
NG3	59.48	5.52	0.33	0.97	42
IDR 2009	35.12	0.00	1.00	1.00	100

Table S11. Description of the selected iLearn descriptors.

Descriptor	Description
GAAC	In the Grouping of Amino Acids According to Composition (GAAC), the 20 types of amino acids are classified into five categories based on their physicochemical properties such as hydrophobicity, charge, and molecular size. The five classes include the aliphatic group (g1: GAVLMI), the aromatic group (g2: FYW), the positively charged group (g3: KRH), the negatively charged group (g4: DE), and the uncharged group (g5: STCPNQ). The GAAC descriptor represents the frequency of each amino acid group.
EGAAC	In the Enhanced Grouping of Amino Acids According to Composition (EGAAC), the GAAC is calculated in fixed-length windows and is generally applied to peptides of equal length.
CKSA-AGP	Amino Acid Group Pairs Spaced k (CKSAAGP) calculates the frequency of pairs of groups $\{g1, g2, g3, g4, g5\}$ of amino acids separated by k residues.
GDPC	Dipeptides by Amino Acid Group Composition (GDPC), $f_{rs} = \frac{N_{rs}}{n-1}, r, s \in \{g1, g2, g3, g4, g5\}$, where N_{rs} is the number of dipeptides in groups r and s.
GTPC	Tripeptides by Amino Acid Group Composition (GTPC) $f_{rst} = \frac{N_{rst}}{n-2}, r, s \in \{g1, g2, g3, g4, g5\}$where N_{rst} is the number of tripeptides in groups r, s, and t.
BLOSUM 62	The BLOSUM62 matrix is used to represent the information of the protein primary sequence as the basic feature set. It uses a matrix composed of $20 \times n$ elements.
CTDC	The Composition descriptor consists of three values: the global compositions (percentage) of the protein's polar, neutral, and hydrophobic residues.
CTDT	The Transition descriptor consists of three values: a transition from the polar group to the neutral group is the percentage frequency with which a polar residue is followed by a neutral residue or a neutral residue by a polar residue. Transitions between the neutral group and the hydrophobic group, as well as between the hydrophobic group and the polar group, are similarly defined.
CTDD	Consists of five values for each of the three groups (polar, neutral, and hydrophobic), i.e., the corresponding fraction of the complete sequence where the first residue of a given group is found, and the remaining 4 values for that group are determined as the corresponding fraction of the sequence where 25%, 50%, 75%, and 100% of the residues for that group are found.

Table S12. Combination of models and parameters update strategies used in every island. (A) Absolute frequencies of appearance across all generations. (B) Absolute frequency of appearance reset every ngr generations. (C) Cumulative value of the function d_{r*} across all generations. (D) Cumulative value of the function d_{r*} reset every ngr generations. Where $d_{r*} = max(1 - \frac{d}{10}, 0)$ and d is the distance between the C_α atoms located at position $r*$ in the reference protein and the designed protein after superposition.

**Parameter update strategy**	**Model 1**	**Model 2**	**Model 3**	**Model 4**	**Model 5**	**Model 6**
A	4	8	12	16	17	20
B	3	7	11	15	18	19
C	2	6	10	14	-	-
D	1	5	9	13	-	-

Table S13. The parameters associated with models. 1 through 4 in each sequence position, and with Bayesian network and Markov chain models.

**Model**	**Parameters**
**1**	$\theta_l^{11r} = P(X = a_l)$
**2**	$\theta_i^{21r} = P(X \in C_{1i}), \theta_j^{22r} = P(X \in C_{3j} \vert C_{1i})$<br>$\theta_k^{23r} = P(X \in C_{4k} \vert C_{1i}, C_{3j}), \theta_l^{24r} = P(X \in a_l \vert C_{1i}, C_{3j}, C_{4k})$
**3**	$\theta_i^{31r} = P(X \in C_{1i}), \theta_o^{32r} = P(X \in C_{2o} \vert C_{1i})$<br>$\theta_j^{33r} = P(X \in C_{3j} \vert C_{1i}, C_{2o}), \theta_l^{34r} = P(X \in a_l \vert C_{1i}, C_{2o}, C_{3j})$
**4**	$\theta_o^{41r} = P(X \in C_{2o}), \theta_i^{42r} = P(X \in C_{1i} \vert C_{2o})$<br>$\theta_j^{43r} = P(X \in C_{3j} \vert C_{1i}, C_{2o}), \theta_l^{44r} = P(X \in a_l \vert C_{1i}, C_{2o}, C_{3j})$
**5* (Bayesian network)**	$\theta_i^{51} = P(X \in a_i), \theta_{ij}^{5q} = p_{ij}^q, p_{ij}^q = P(X = a_j \vert X = a_i)$, for the arc *q*,<br>$P_{ij}^q = \{p_{ij}^q\}$ is the transition matrix for the arc *q*.
**6* (Markov chain)**	$\theta_i^{61} = P(X \in a_i), \theta_{ij}^{6q} = p_{ij}^{q\infty}, p_{ij}^{q\infty} = P(X = a_j \vert X = a_i)$ when time tends to infinity<br>$P_{ij}^{q\infty} = \{p_{ij}^{q\infty}\}$ is the transition matrix

$l = 1, \ldots, 20$, $i = 1, \ldots, 3$, $j = 1, \ldots, 6$, $k = 1, \ldots, 15$, $o = 1, \ldots, 4$, $r = 1, \ldots, n$, position in the sequence.

$* \, i = 1, \ldots, 20, j = 1, \ldots, 20, q = 1, \ldots, (n-1)$

Table S14. Proteins selected for design using our algorithm. PDB id.: four-letter code of the protein in the PDB, Sequence: the subsequence corresponding to the selected structure, n: length of the selected subsequence, Protein name: the name of the protein in the PDB, 2D: the corresponding secondary structure, ha: alpha helix, lb: beta sheets, nd: not defined.

PDB ID	Sequence	n	Protein name (from PDB)	2D
1EMN	GQCINTDGSYRCECPFGYILAGNECVD	27	Fibrillin-1	lb
1LQL	KYDITAVLNED SSMTAISDQFQITLD	26	Hydroperoxide reductase	lb
1NP6	ELRKAGAAQTI VASQQRWALMTETPDE	27	Molybdopterin-guanine dinucleotide biosynthesis adapter protein	lb
1O6W	WKEAKDASGRIY YYNTLTKKSTWEKP	26	Pre-mRNA-processing protein PRP40	lb
2E45	GWMRVQDTSGT YYWHIPTGTTQW	23	Amyloid beta precursor protein binding family B member 1	lb
2KXQ	LPEGYEQRTTQQG QVYFLHTQTGVSTWHDP	30	E3 ubiquitin-protein ligase SMURF2	lb
1S7M	EVKFKSGNGINV SGKTVNGRREITFELA	28	Adhesin	lb
1P9N	DSYELRKAGAAQTIV ASQQRWALMTETPDE	33	Molybdopterin-guanine dinucleotide biosynthesis adapter protein	lb
5UIY	HALVFSYISFALISDKRYQ	19	Bromodomain adjacent to zinc finger domain protein 1A	ha
3CLQ	APTLYEKIQQANEEAVTRIIQS	22	Enterococcus faecalis V583 Uncharacterized protein	ha
3SB1	SPRADVAAARAALVLRMADL	20	HupH hydrogenase expression protein C-terminal domain-containing protein	ha
2QQ8	YFQGNAVLTWNNEILPNWE	19	TBC1 domain family member 14	ha
3M9Q	TEENRQLQRELAEAAKL	17	Protein male-specific lethal-3	ha
3H25	GPALVQQAGQQIEQAQRQQE	20	Replication protein B	ha
3EWK	KEAEAQLARLKQAMDAN	17	Histidine kinase	ha
3C8V	GGPFLDSFKKRIKRILSEN	19	Hexapeptide repeat-containing transferase	ha
2QIW	PLWQKWLAATSAQQLKGWA	19	PEP phosphonomutase and related enzymes	ha
2OAR	TQVVLLTEIRDLLAQTN	17	Large-conductance mechanosensitive channel	ha
2LKM	PQLRRPFELLIAAAMER	17	PHD finger protein 12	ha
1MSL	GEVEQPGDTQVVLLTEIR	18	Large-conductance mechanosensitive channel	ha
3W68	PELSADLRPRSILGL	15	Alpha-tocopherol transfer protein	nd
1R5L	PEISADLHPRSIIGL	15	Alpha-tocopherol transfer protein	nd
1N7D	RQFVCDSDRDCLDGSDEASCPVLTCGPA	28	Extracellular domain of the LDL receptor	nd

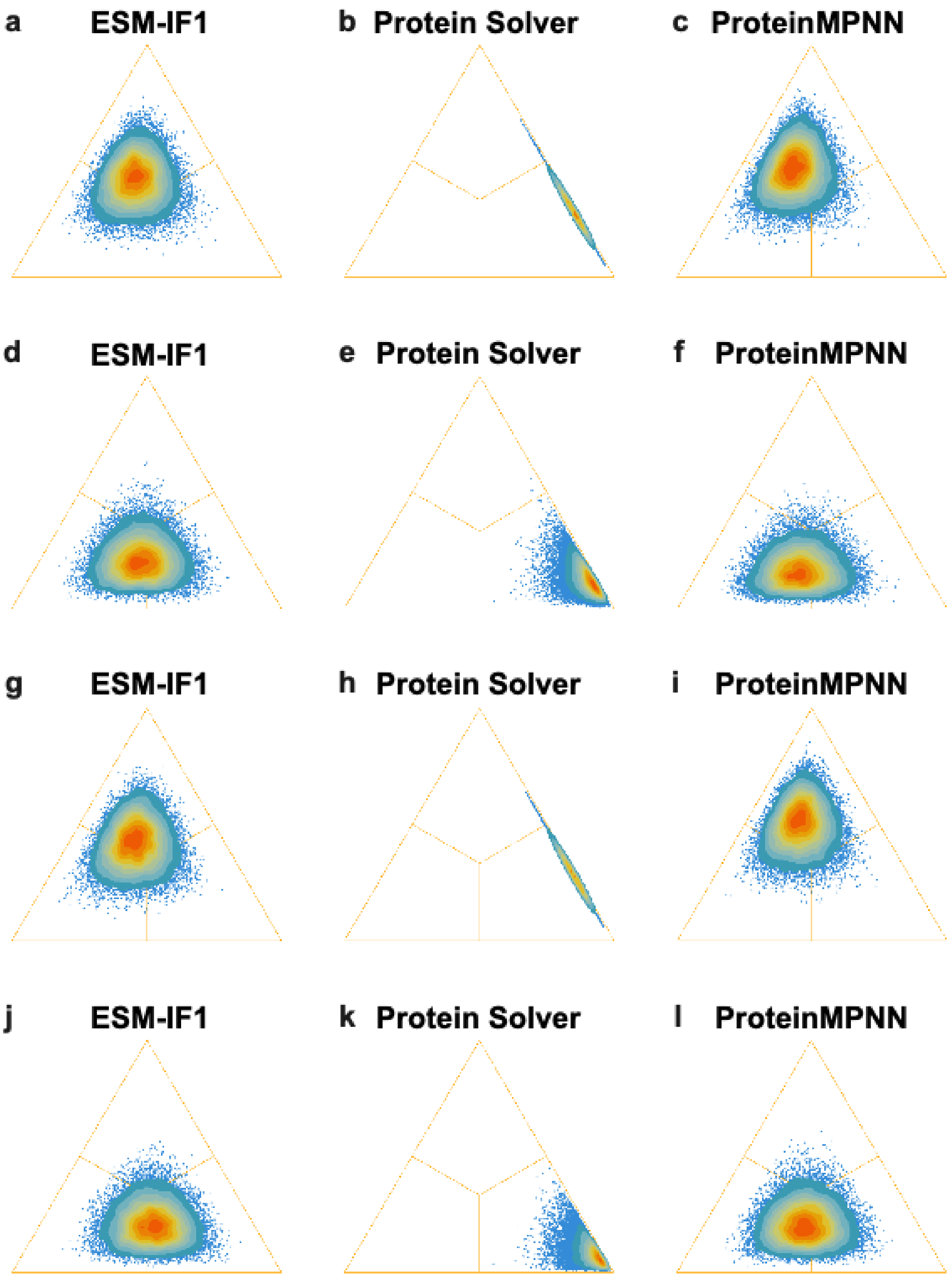

Figure S1. **Sign test and Bayesian paradigm, comparison between KCM, ESM-IF1, Protein Solver, and ProteinMPNN. (a-c)** Comparison of GDT_TS of KCM with respect to **(a)** ESM-IF1, **(b)** Protein Solver, and **(c)** ProteinMPNN, when analyzing the fittest 50 solutions obtained for

each protein in each algorithm. **(d-f)** Comparison of RMSD of KCM with respect to **(d)** ESM-IF1, **(e)** Protein Solver, and **(f)** ProteinMPNN, when analyzing the fittest 50 solutions obtained for each protein in each algorithm. **(g-i)** Comparison of GDT_TS of KCM with respect to **(g)** ESM-IF1, **(h)** Protein Solver, and **(i)** ProteinMPNN, when analyzing the fittest 250 solutions obtained for each protein in each algorithm. **(j-l)** Comparison of RMSD of KCM with respect to **(j)** ESM-IF1, **(k)** Protein Solver, and **(l)** ProteinMPNN, when analyzing the fittest 250 solutions obtained for each protein in each algorithm. If the points are concentrated on the left lower corner means that KCM loses, if they are concentrated on the right lower corner then it means that KCM wins. If the points are concentrated on the upper part, both methods perform equally.

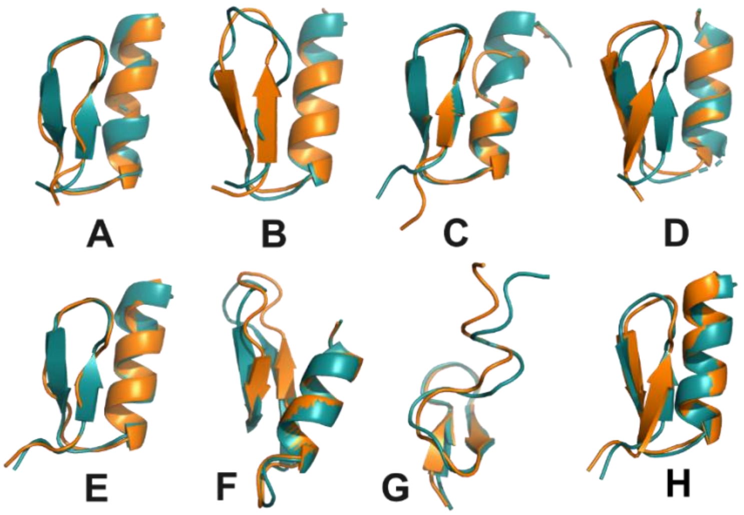

Figure S2. Backbone superposition of the designed α/β-proteins with their references. Designed sequences are colored orange, while reference structures are colored green. A-1AAY, B-1F2I, C-1LLM, D-2I13, E-2PRT, F-3E7R, G-3VDU, H-4GZN.

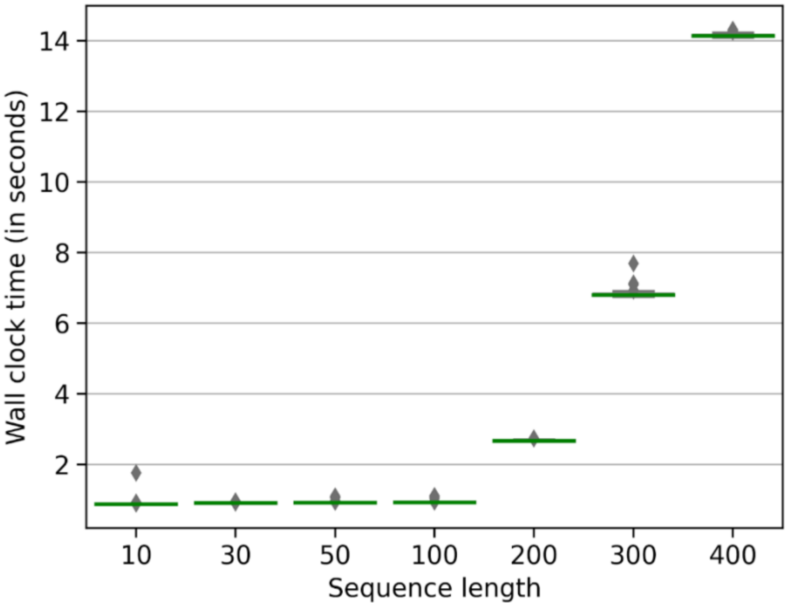

Figure S3. Box plot showing the runtime of ESMFold as a function of sequence length. Each box represents the distribution of runtimes for 100 randomly generated sequences of a given length. Green horizontal lines denote the mean runtime, while gray diamonds indicate outlier values. This analysis illustrates the computational cost of structure prediction as sequence length increases.

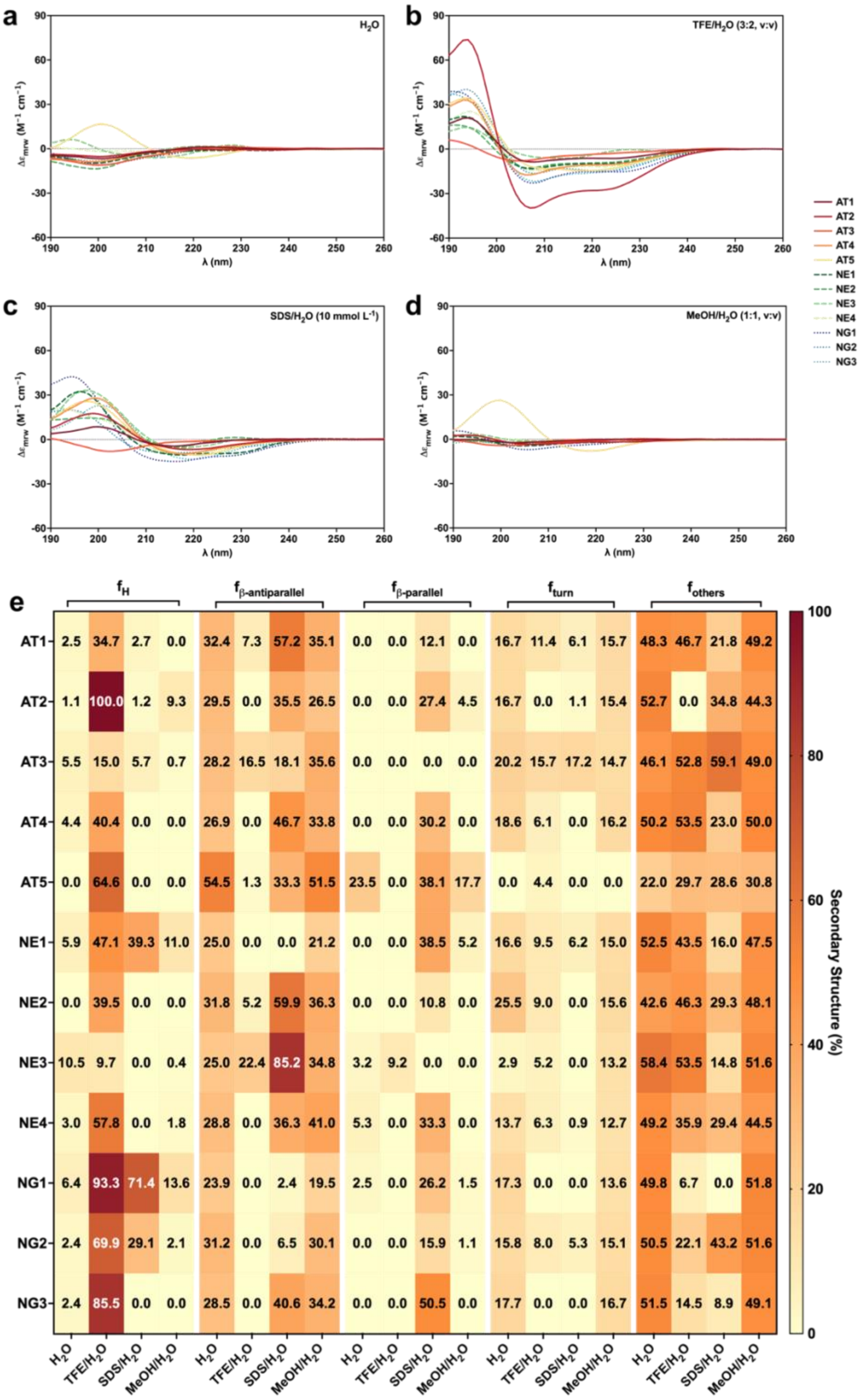

Figure S4. Circular dichroism spectra of designed peptides. Circular dichroism experiments were conducted with the peptides using a J-1500 Jasco circular dichroism spectrophotometer. The spectra were recorded in four different media: **(a)** water, **(b)** 60% trifluoroethanol in water, **(c)** sodium dodecyl sulfate (SDS) in water (10 mmol L^{-1}), and **(d)** 50% methanol in water, after three accumulations at 25 ^{o}C, using a 1mm path length quartz cell, between 260 and 190 nm at 50 nm min^{-1}, with a bandwidth of 0.5 nm. The concentration of all peptides tested was 50 μmol L^{-1}. **(e)** Heatmap with the percentage of secondary structure found for each peptide in the four different media analyzed. Secondary structure fraction was calculated using the BeStSel server[7].

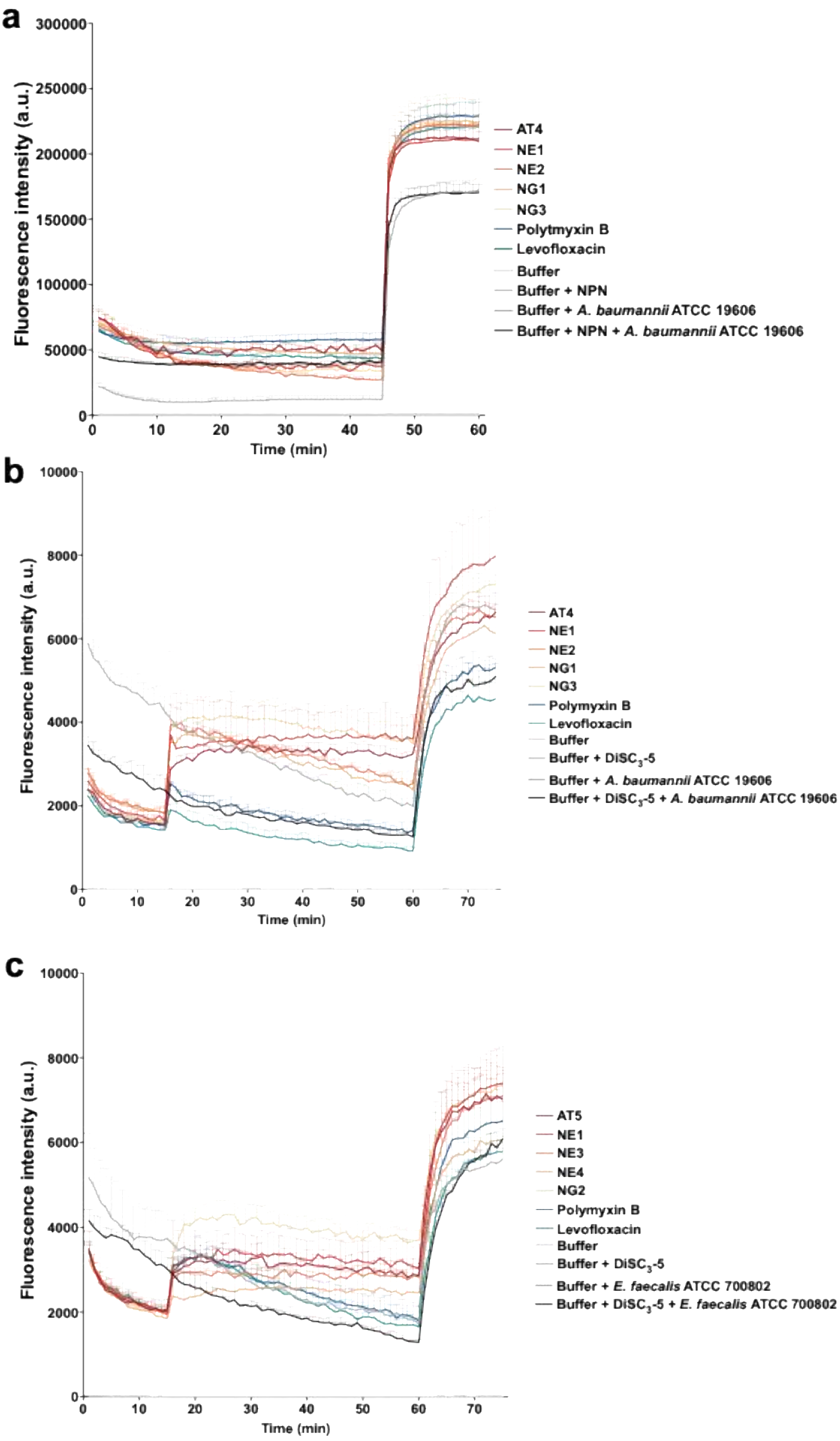
a
Fluorescence intensity (a.u.)
300000
250000
200000
150000
100000
50000
0
Time (min)
AT4
NE1
NE2
NG1
NG3
Polytmyxin B
Levofloxacin
Buffer
Buffer + NPN
Buffer + A. baumannii ATCC 19606
Buffer + NPN + A. baumannii ATCC 19606
b
Fluorescence intensity (a.u.)
Time (min)
AT4
NE1
NE2
NG1
NG3
Polymyxin B
Levofloxacin
Buffer
Buffer + DiSC3-5
Buffer + A. baumannii ATCC 19606
Buffer + DiSC3-5 + A. baumannii ATCC 19606
c
Fluorescence intensity (a.u.)
Time (min)
AT5
NE1
NE3
NE4
NG2
Polymyxin B
Levofloxacin
Buffer
Buffer + DiSC3-5
Buffer + E. faecalis ATCC 700802
Buffer + DiSC3-5 + E. faecalis ATCC 700802

Figure S5. Outer membrane permeabilization and cytoplasmic membrane depolarization of *A. baumannii* ATCC 19606 induced by designed peptides. (a) Outer membrane permeabilization was assessed using the probe 1-(N-phenylamino)naphthalene (NPN), showing the permeabilization effects of the designed peptides active against *A. baumannii* ATCC 19606. **(b)** Membrane depolarization assays were performed using the hydrophobic probe 3,3′-dipropylthiadicarbocyanine iodide [DiSC$_3$-(5)] on all active peptides against *A. baumannii* ATCC 19606 and vancomycin-resistant *E. faecalis* ATCC 700802. Polymyxin B and levofloxacin served as positive controls, while buffer, buffer with the probe, and buffer with both probe and bacteria were used as baseline controls for fluorescence. The panels display the raw fluorescence intensity data obtained from the experiments. Error bars are the standard deviation obtained from the three replicates.

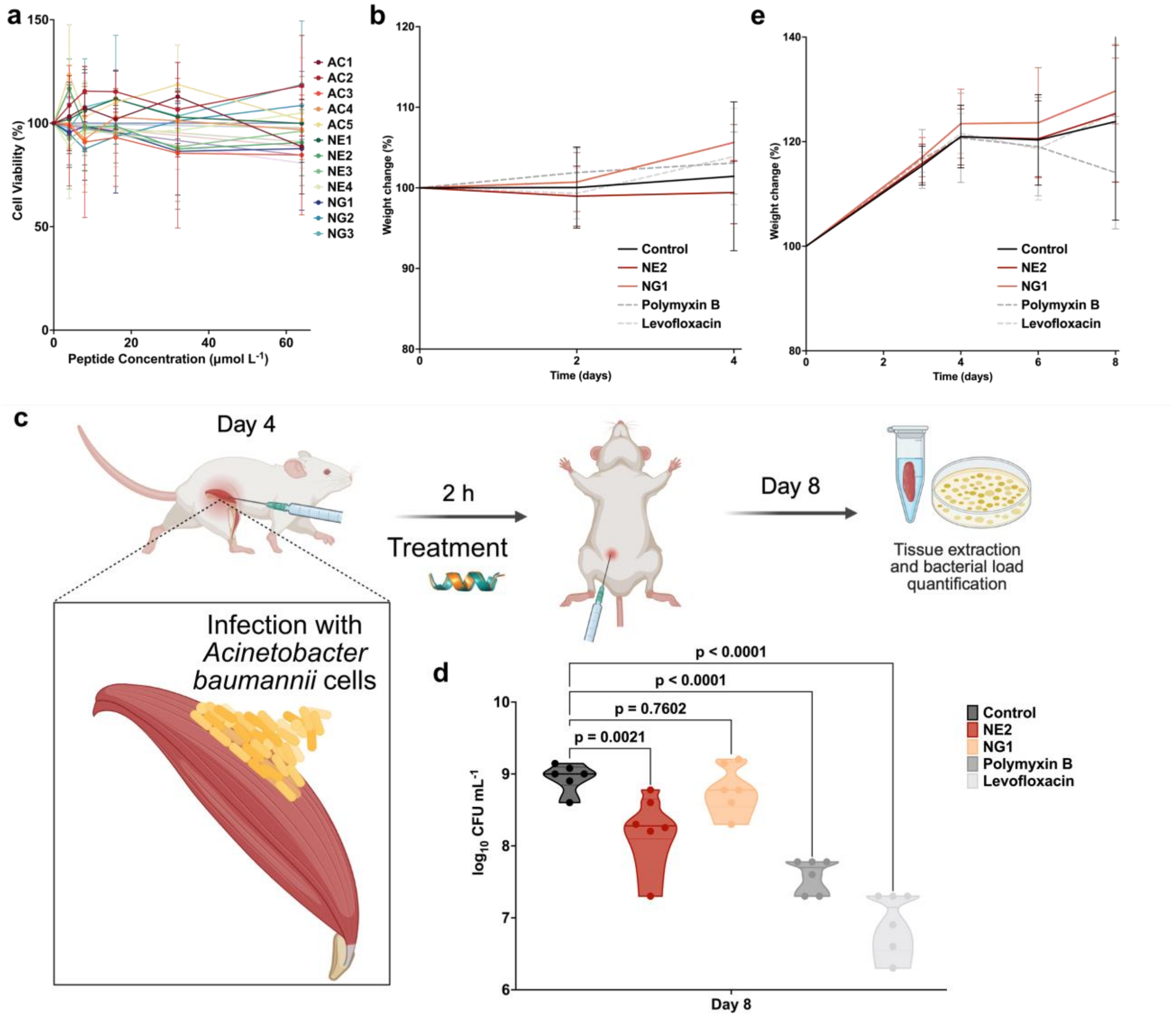

Figure S6. Cytotoxicity and anti-infective activity of designed peptides. (a) Cytotoxic effects of increasing concentrations (4, 8, 16, 32, and 34 μmol L^{-1}) of each peptide on human embryonic kidney (HEK293T) cells after 24 h of treatment. Mouse weight was monitored throughout the duration of the **(b)** skin abscess model (4 days total) and the **(e)** deep thigh infection model (8 days total) to assess potential toxic effects of both the bacterial load and the designed peptides. **(c)** Schematic of the neutropenic thigh infection mouse model, where the designed peptides were administered intraperitoneally. Anti-infective activity against *A. baumannii* ATCC 19606 was evaluated 4 days after intraperitoneal peptide administration (n = 6). **(d)** Four days after intraperitoneal injection, NE2 at its MIC (32 μmol L^{-1}) inhibited by one order of magnitude the *A. baumannii* ATCC19606 infection, though its activity was less potent than that of polymyxin B and levofloxacin, compared to the untreated control group. Statistical significance in panel **d** was determined using one-way ANOVA followed by Dunnett's test; P values are shown in the graphs. In the violin, the center line represents the mean, the box limits the first and third quartiles, and the whiskers (minima and maxima) represent 1.5 × the interquartile range. The solid line inside each box represents the mean value obtained for each group. Error bars in **a**, **b** and **e** are the standard deviation obtained from the three biological replicates. Each group consisted of six mice (n = 6). Panel **c** was created with BioRender.com. De La Fuente-Nunez, C. (2025) https://BioRender.com/5dpvmsp.
