# Supplementary material for: Tailored structured peptide design with a key-cutting machine approach
Source: Nat Mach Intell. 2025 Oct 21;7(10):1685–97. doi: 10.1038/s42256-025-01119-2 (PMC12552119; doi:10.1038/s42256-025-01119-2)
Supplement: Supplementary file 2 — Reporting Summary [file 42256_2025_1119_MOESM2_ESM.pdf]

Corresponding author(s): NATMACHINTELL-A25020499A

Last updated by author(s): Aug 5, 2025

Reporting Summary

Nature Portfolio wishes to improve the reproducibility of the work that we publish. This form provides structure for consistency and transparency in reporting. For further information on Nature Portfolio policies, see our Editorial Policies and the Editorial Policy Checklist.

Statistics

For all statistical analyses, confirm that the following items are present in the figure legend, table legend, main text, or Methods section.

n/a	Confirmed	
☐	☒	The exact sample size (*n*) for each experimental group/condition, given as a discrete number and unit of measurement
☒	☐	A statement on whether measurements were taken from distinct samples or whether the same sample was measured repeatedly
☐	☒	The statistical test(s) used AND whether they are one- or two-sided<br>*Only common tests should be described solely by name; describe more complex techniques in the Methods section.*
☒	☐	A description of all covariates tested
☐	☒	A description of any assumptions or corrections, such as tests of normality and adjustment for multiple comparisons
☐	☒	A full description of the statistical parameters including central tendency (e.g. means) or other basic estimates (e.g. regression coefficient) AND variation (e.g. standard deviation) or associated estimates of uncertainty (e.g. confidence intervals)
☐	☒	For null hypothesis testing, the test statistic (e.g. F, t, r) with confidence intervals, effect sizes, degrees of freedom and P value noted<br>*Give P values as exact values whenever suitable.*
☒	☐	For Bayesian analysis, information on the choice of priors and Markov chain Monte Carlo settings
☒	☐	For hierarchical and complex designs, identification of the appropriate level for tests and full reporting of outcomes
☐	☒	Estimates of effect sizes (e.g. Cohen's *d*, Pearson's *r*), indicating how they were calculated

Our web collection on statistics for biologists contains articles on many of the points above.

Software and code

Policy information about availability of computer code

Data collection	No software was used for data collection.
Data analysis	Figure 1, panels (b) and (c) were created using R version 4.5.0.<br>Figure 1, panel (b) was created using the ggridge package, version 0.5.6, by means of the geom_density_ridges function.<br>Figure 1, panel (c) was created using the ggplot2 package, version 3.5.2, by means of the geom_point function.<br>Figure 1, panel (d) was created using PyMOL, version 2.5.0, by means of the super command.<br>All experimental results were analyzed using GraphPad Prism, version 10.4.1. Single spectrum analysis tool of BeStSel was used to analyze the circular dichroism spectra (https://bestsel.elte.hu/index.php).

For manuscripts utilizing custom algorithms or software that are central to the research but not yet described in published literature, software must be made available to editors and reviewers. We strongly encourage code deposition in a community repository (e.g. GitHub). See the Nature Portfolio guidelines for submitting code & software for further information.

Data

Policy information about availability of data

All manuscripts must include a data availability statement. This statement should provide the following information, where applicable:

- Accession codes, unique identifiers, or web links for publicly available datasets
- A description of any restrictions on data availability
- For clinical datasets or third party data, please ensure that the statement adheres to our policy

This study did not generate new unique reagents. Further information and requests for resources should be directed to the lead contact, Carlos A. Brizuela (cbrizuel@cicese.mx), and will be fulfilled upon reasonable request.

Research involving human participants, their data, or biological material

Policy information about studies with human participants or human data. See also policy information about sex, gender (identity/presentation), and sexual orientation and race, ethnicity and racism.

Reporting on sex and gender	n/a
Reporting on race, ethnicity, or other socially relevant groupings	n/a
Population characteristics	n/a
Recruitment	n/a
Ethics oversight	n/a

Note that full information on the approval of the study protocol must also be provided in the manuscript.

Field-specific reporting

Please select the one below that is the best fit for your research. If you are not sure, read the appropriate sections before making your selection.

☒ Life sciences ☐ Behavioural & social sciences ☐ Ecological, evolutionary & environmental sciences

For a reference copy of the document with all sections, see nature.com/documents/nr-reporting-summary-flat.pdf

Life sciences study design

All studies must disclose on these points even when the disclosure is negative.

Sample size	Sample sizes were calculated on the basis of previous literature on similar experimental settings (Wan and Torres et al., 2024. DOI: 10.1038/s41551-024-01201-x; Guan and Torres et al., 2025. DOI: 10.1038/s41467-025-60051-6; Torres et al., 2024. DOI: 10.1016/j.cell.2024.07.027).
Data exclusions	No data was excluded.
Replication	Bacterial growth inhibition, cytotoxicity, permeabilization, and depolarization assays were all measured in 3 individual biological replicates. All attempts of replication were successful.
Randomization	For bacterial growth inhibition and cytotoxicity assays, peptides were plated in a random order. Mice and cages were randomly allocated to the experimental groups.
Blinding	Peptides were solubilized and labelled with internal codes for completely unbiased experimentation by the same researcher.

Reporting for specific materials, systems and methods

We require information from authors about some types of materials, experimental systems and methods used in many studies. Here, indicate whether each material, system or method listed is relevant to your study. If you are not sure if a list item applies to your research, read the appropriate section before selecting a response.

Materials & experimental systems

n/a	Involved in the study	
☒	☐	Antibodies
☐	☒	Eukaryotic cell lines
☒	☐	Palaeontology and archaeology
☐	☒	Animals and other organisms
☒	☐	Clinical data
☒	☐	Dual use research of concern
☒	☐	Plants

Methods

n/a	Involved in the study	
☒	☐	ChIP-seq
☒	☐	Flow cytometry
☒	☐	MRI-based neuroimaging

Eukaryotic cell lines

Policy information about cell lines and Sex and Gender in Research

Cell line source(s)	Human embryonic kidney (HEK293T) cells were obtained from the American Type Culture Collection (ATCC; CRL-3216™).
Authentication	None of the cell lines used were authenticated.
Mycoplasma contamination	The cell lines were not tested for mycoplasma contamination.
Commonly misidentified lines (See ICLAC register)	No commonly misidentified cell lines were used in this study.

Animals and other research organisms

Policy information about studies involving animals; ARRIVE guidelines recommended for reporting animal research, and Sex and Gender in Research

Laboratory animals	CD-1 mice (4-6 weeks old) from Charles River Laboratory (catalog number 18679700-022).
Wild animals	No wild animals were used in this study.
Reporting on sex	All mice used in the experiments were female.
Field-collected samples	No field-collected samples were used in this study.
Ethics oversight	The animal model protocols skin abscess infection mouse model (806763) and thigh infection model (807055) were revised and approved by the University Laboratory Animal Resources (ULAR) from the University of Pennsylvania.

Note that full information on the approval of the study protocol must also be provided in the manuscript.

Plants

Seed stocks	n/a
Novel plant genotypes	n/a
Authentication	n/a
